# Complex approach for analysis of snake venom α-neurotoxins binding to HAP, the high-affinity peptide

**DOI:** 10.1038/s41598-020-60768-y

**Published:** 2020-03-02

**Authors:** Denis S. Kudryavtsev, Valentin М. Tabakmakher, Gleb S. Budylin, Natalia S. Egorova, Roman G. Efremov, Igor A. Ivanov, Svetlana Yu. Belukhina, Artjom V. Jegorov, Igor E. Kasheverov, Elena V. Kryukova, Irina V. Shelukhina, Evgeny A. Shirshin, Nadezhda G. Zhdanova, Maxim N. Zhmak, Victor I. Tsetlin

**Affiliations:** 1 Shemyakin-Ovchinnikov Institute of Bioorganic Chemistry, Russian Academy of Sciences, Moscow, 117997 Russia; 2 School of Biomedicine, Far Eastern Federal University, Vladivostok, 690950 Russia; 3 Faculty of Physics, National Research University Higher School of Economics, Moscow, 101000 Russia; 4 National Research University Higher School of Economics, Moscow, 101000 Russia; 5 Moscow Institute of Physics and Technology (State University), Dolgoprudny, 141701 Moscow Oblast, Russia; 6 Faculty of Biotechnology, Moscow State University, Moscow, 119991 Russia; 7 Sechenov First Moscow State Medical University, Institute of Molecular Medicine, Moscow, 119991 Russia; 8 Department of Physics, M.V. Lomonosov Moscow State University, Moscow, 119992 Russia; 9 Institute of spectroscopy of the Russian Academy of Sciences, Troitsk, Moscow, 108840 Russia; 10 PhysBio of MePhi, 115409 Moscow, Russia

**Keywords:** Protein design, Molecular modelling, Biological fluorescence, Peptides, Ligand-gated ion channels

## Abstract

Snake venom α-neurotoxins, invaluable pharmacological tools, bind with high affinity to distinct subtypes of nicotinic acetylcholine receptor. The combinatorial high-affinity peptide (HAP), homologous to the C-loop of α1 and α7 nAChR subunits, binds biotinylated α-bungarotoxin (αBgt) with nanomolar affinity and might be a protection against snake-bites. Since there are no data on HAP interaction with other toxins, we checked its binding of α-cobratoxin (αCtx), similar to αBgt in action on nAChRs. Using radioiodinated αBgt, we confirmed a high affinity of HAP for αBgt, the complex formation is supported by mass spectrometry and gel chromatography, but only weak binding was registered with αCtx. A combination of protein intrinsic fluorescence measurements with the principal component analysis of the spectra allowed us to measure the HAP-αBgt binding constant directly (29 nM). These methods also confirmed weak HAP interaction with αCtx (>10000 nM). We attempted to enhance it by modification of HAP structure relying on the known structures of α-neurotoxins with various targets and applying molecular dynamics. A series of HAP analogues have been synthesized, HAP[L9E] analogue being considerably more potent than HAP in αCtx binding (7000 nM). The proposed combination of experimental and computational approaches appears promising for analysis of various peptide-protein interactions.

## Introduction

α-Neurotoxins are snake venom proteins serving as accurate tools in research on nicotinic acetylcholine receptors (nAChRs) (see reviews^1–3^). Their valuable feature is the capacity to distinguish certain nAChRs subtypes: short-chain α-neurotoxins bind only to the muscle-type nAChRs, while the long ones, such as α-bungarotoxin (αBgt) and α-cobratoxin (αCtx), are blocking muscle-type nAChRs and the neuronal ones containing α7, α9 and α9/α10 subunits^4–6^. The spatial structure of all α-neurotoxins consists of three β-structural loops, giving them the name of “three-finger” proteins (with the abbreviations 3FP or TFPs), their arrangement being maintained by 4 disulfide bridges in the short and by 5 disulfides in the long α-neurotoxins. Interestingly, the TFP family embraces not only numerous α-neurotoxins, but also diverse snake venom proteins acting specifically on different targets (ion channels, G-protein coupled receptors, enzymes) or, in the case of cytotoxins, just disrupting cell membranes (see^7^). The interest to α-neurotoxins in recent years was invigorated by the discovery that some so-called Ly6 proteins, which also have a three-finger folding and are present in a wide range of organisms from *Drosophila* to human beings, interact with nAChRs (see reviews^1,8^). Such Ly6 proteins as Lynx1 (attached to the membrane by glycosylphosphatidylinositol anchor) or SLURP1 (secreted protein) are endogenous regulators of the physiological and pathophysiological functions of nAChRs and design of novel drugs on their basis requires an understanding of mechanisms of their action. Here only first steps were made (see recent references in^9^), and we believe that the assistance may come from research on α-neurotoxins for which a wealth of information is available, including comprehensive analysis of their interactions with nAChRs (mutations and electrophysiology), as well as the X-ray and NMR structures for α-neurotoxin complexes with the nAChR models.

The best nAChR models are water-soluble acetylcholine-binding proteins (AChBPs). The X-ray structure of AChBP for the first time demonstrated how a ligand-binding domain (LBD) of the nAChR should look^10,11^. Then followed the X-ray structures of AChBP complexes with various agonists and antagonists (see review^12^), including those with α-neurotoxins and α-conotoxins, as well as the αBgt complexes with the ligand-binding domains of the nAChR α1 and α9 subunits^13,14^. These structures were utilized to deduce information about the binding sites of distinct whole-size nAChRs.

The role of the nAChR models or “mimics” to a certain extent can be played by relatively short peptides. Indeed, α-neurotoxins for a long time were known to bind to synthetic fragments of nAChR α1 and α7 subunits encompassing their C-loops, as well as to the combinatorial peptides sharing a certain similarity with this loop^15^. The affinity of such binding may be sufficiently high, and recently this property was utilized by introducing the respective sequences into the extracellular parts of diverse receptors, differing from the nAChRs. Fluorescent or biotinylated αBgt derivatives made possible detecting of such receptors in the cells and monitoring receptor assembly and cell internalization; among the successful examples were such ion channels as α-amino-3-hydroxy-5-methyl-4-isoxazolepropionic acid (AMPA) receptors, ionotropic γ-aminobutiryc acid (GABAA) receptors and voltage-gated potassium Kv4.2 channels, as well as such G-protein coupled receptors as γ-aminobutiryc acid receptors (GABAB)^16^.

Our attention was attracted to a 13-membered peptide HAP (“high affinity peptide”), which binds biotinylated αBgt with a high affinity (2–4 nM), characteristic for the αBgt binding to the whole-size nAChRs. The X-ray structure of HAP in complex with αBgt and ^1^H-NMR structure of a close HAP homolog bound to αBgt have been solved^17,18^. Such high affinity stimulated the idea that HAP might be used as a remedy against snake bites. The principle possibility of such an approach against poisoning was recently shown by application of AChBP which could bind long, but not short α-neurotoxins^19,20^. This line of research is still of interest. Previously, only the interaction of HAP with biotinylated αBgt has been described. One of the purposes of our work was to check how HAP interacts with a different long-chain α-neurotoxin, namely α-cobratoxin from the *Naja kaouthia* which binds to nAChRs almost with the same affinity as αBgt. In competition with radioiodinated αBgt we confirmed the earlier reported high affinity binding of HAP to biotinylated αBgt. However, we registered only very weak binding to αCtx. It prompted us to analyze available data on the spatial structures of αBgt and αCtx in complexes with the peptide fragments and nAChR models and to design a HAP analog with the increased affinity for αCtx. For this purpose we combined computational approaches, radiolgand analysis, gel chromatography, mass spectrometry and fluorescence spectroscopy for a thorough analysis of interactions between HAP and its analogs with αBgt and αCtx. Although for αCtx we succeeded in only moderate increase in the affinity, we hope that the proposed approach will be helpful for analysis of various peptide-protein interactions.

## Materials and methods

### Synthesis of HAP and its analogs

Solid-phase synthesis utilizing the Fmoc strategy was performed to make the peptides. The first amino acid residue was attached to Wang-type polystyrene resin (Iris Biotech, Germany) by symmetric anhydride method. Polypeptide chain assembly was performed on Syro II automatic peptide synthesizer (MultiSynTech AG, Germany) with DIC/HOAt activation.

Preparative purification was carried out as described previously^21^ using a Gilson HPLC system (333/334 pump with 215 liquid handler) equipped with a YMC Triart 10um 30 × 150 mm column and UV detection at 210 and 280 nm. Peptides elution was achieved by addition of a H_2_O-acetonitrile in the 10–55% gradient with 0.1% v/v CF3COOH. HPLC-MS analysis was performed using Thermo Finnigan LCQ Deca XP ion trap instrument with Thermo Accela UPLC system equipped with Waters Atlantis T3 3um 150 × 2 mm column. Detection was achieved by UV-VIS DAD and full scan MS (ESI+, 150–2000 Th).

### Radioligand analysis of the HAP and its analogs interactions with αBgt and αCtx

Competition of HAP and its analogues with mono-iodinated ^125^I-αBgt and ^125^I-αCtx^22^ (500 Ci/mmol) was carried out on two targets - muscle-type nAChR in membrane preparation of *Torpedo californica* ray electric organ and neuronal human α7 nAChR transfected in GH4C1 cell line. For competitive radioligand assays, suspensions of *T*. *californica* nAChR-rich membranes (1.25 nM α-bungarotoxin binding sites, kindly provided by Prof. F. Hucho, Institute for Chemistry and Biochemistry, Freie Universität Berlin, Germany) in 50 μL of 20 mM Tris-HCl buffer pH 8.0, containing 1 mg/ml BSA (binding buffer) or human α7 nAChR transfected cells (0.4 nM α-bungarotoxin binding sites, received from Eli Lilly and Company, London, UK) in 50 μL of binding buffer were incubated for 3 h at room temperature with various amounts of HAP or its analogues, followed by an additional 5 min incubation with 0.5–0.9 nM of radioligands. Nonspecific binding was determined by preliminary incubation of the preparations with 30 µM α-cobratoxin. The membrane and cell suspensions were applied to glass GF/C filters (Whatman, Maidstone, UK) presoaked in 0.25% polyethyleneimine, and unbound radioactivity was removed from the filter by washing (3 × 3 ml) with 20 mM Tris-HCl buffer, pH 8.0 containing 0.1 mg/ml BSA (washing buffer). The bound radioactivity was determined using a Wizard 1470 Automatic Gamma Counter^23^.

### Computer modeling

The experimentally determined structure (atomic coordinates) of αBgt complexes with HAP and HAP[L9E] were obtained from the RCSB Protein Data Bank (PDB ID: 1HC9, 1HAJ, respectively)^17,18^. Structure of αCtx was derived from its complex with *Ls*-AСhBP (PDB ID: 1YI5^24^). The models of αCtx complexes with HAP and HAP[L9E] were built on the basis of complexes with αBgt (PDB ID: 1HC9 and 1HAJ) by 3D alignment αCtx onto αBgt and subsequent removal of αBgt. Models of HAP variants with point amino acid substitutions complexed to αCtx and αBgt were built using *in silico* mutagenesis in the PyMOL program (The PyMOL Molecular Graphics System, Version 1.8 Schrödinger, LLC.).

### Molecular dynamics simulations

Molecular dynamics (MD) simulations of free HAP, HAP[L9E] and other point mutation variants of HAP peptide, free α-neurotoxins, as well as their complexes, were performed in explicitly defined water environment with the GROMACS 5.1.2 software^25^ using the Gromos96 43a3 parameters set^26^. Peptides’ complexes with α-neurotoxins were solvated inside (6.0–6.7 nm)^3^ cubic boxes with 6900–9560 water molecules; the SPC water model^27^ was used; the required number of Cl^−^ or Na^+^ ions to maintain electroneutrality was added. Analogously, free HAP and HAP[L9E] peptides and free α-neurotoxins were solvated in (4.32–4.42 nm)^3^/(6.28–6.76 nm)^3^ boxes with 2535–2700/7660–9615 water molecules. Simulations were carried out with a time step of 2 fs, imposing 3D periodic boundary conditions, in the isothermal-isobaric (NPT) ensemble using a Berendsen barostat^28^ (pressure of 1 bar) and a V-rescale thermostat^29^ (temperature of 37 °C). Van der Waals interactions were truncated using a 1.2 nm spherical cut-off function. Electrostatic interactions were treated with the PME algorithm. The length of MD trajectories of toxin–peptide complexes and free α-neurotoxins was 200 ns, which is enough to sample internal conformational movements and account inter- and intramolecular contacts for this size molecular system. The length of the MD trajectories of free peptides was 500 ns.

### Computational analysis of peptides and their complexes with α-neurotoxins

Analysis of inter- and intramolecular contacts in MD trajectories and interaction energy profiles evaluation was performed using an in-house software package IMPULSE [Krylov *et al*., in preparation] analogously to the procedures described in our previous study^30^.

H-bonds were assigned using parameters set from hbond utility of GROMACS software^25^ (distance D—A ≤ 3.5 Å and angle D—H—A ≥ 150° for the hydrogen bond D—H•••A); salt bridges, π-cation, stacking, and hydrophobic contacts were calculated using algorithms described in our previous works^31,32^. All drawings showing 3D structures were prepared with the PyMOL Molecular Graphics System, version 1.8 (The PyMOL Molecular Graphics System, Version 1.8 Schrödinger, LLC.).

Gromos96 43a3 parameters set^26^ and 1.2 nm cutoff distance for Lennard-Jones/electrostatic interactions were used during the intermolecular short-range non-bonded interaction energy estimation. The latter being the sum of the Lennard-Jones and electrostatic terms. Graphical representation of interaction energy profiles was performed using Python built-in libraries and NumPy package.

Investigation of the molecular surface was performed using Naccess program^33^ and the technique described elsewhere^34^.

### Steady-state fluorescence

The titration series for each peptide-toxin pair were performed to analyze the interaction of toxins and peptides. Each titration series was obtained by the addition of small volumes of the peptide to the stock solution of toxin, and tryptophan (Trp) fluorescence spectra at 280 nm excitation were obtained for each sample.

Fluorescence spectra were measured using Fluoromax-4 (Horiba Jobin Yvon). The excitation wavelength was set as λ_exc_ =280 nm to excite proteins intrinsic fluorescence. Fluorescence emission was measured in the 300–500 nm wavelength region, the bandwidths of both slits were set to 2 nm. All measurements were performed at room temperature (25 ± 1 °C) without temperature stabilization.

The stock solutions for fluorescence measurements were prepared using Tris-HCl buffer (pH 7.40 ± 0.03, 50 mM, [NaCl] = 0.1 M). Concentrations of stock solutions of peptides and toxins were determined spectrophotometrically using known extinction coefficients at 280 nm. Spectra were subjected to the principal component analisys and fitted to the equations:

1. K_d_ = [α-neurotoxin][binding peptide]/[Complex],
2. [α-neurotoxin]_total_ = [α-neurotoxin] + [Complex],
3. [binding peptide]_total_ = [binding peptide] + [Complex],

where K_d_ is the dissociation constant (affinity), and equilibrium concentrations of each component are shown in square brackets.

Data processing, fitting and plotting, were performed using Python programming language and NumPy, lmfit and matplotlib libraries.

### Liquid chromatography

Superdex 75 10/300 GL column with AKTA Purifier (GE Healthcare Bio-Sciences, PA, USA) was utilized to determine tested peptide complex formation with the αBgt or αCtx using the following protocol: 0.1 M ammonium acetate solution (pH 6.2), flow speed set to 0.5 ml/min, optical density detection at 226 nm and 280 nm. Binding reactions performed in 20 mM Tris-HCl pH 8.0 with 30 µМ αBgt or αCtx and various concentrations of HAP or HAP[L9E] peptides (10 µМ, 20 µМ, 30 µМ, 40 µМ, 60 µМ). Chromatography results were quantified using UNICORN Manager program. HPLC-MS analysis of fractionated peaks was performed using Thermo Finnigan LCQ Deca XP ion trap instrument with Thermo Accela UPLC system equipped with Waters Atlantis T3 3um 150 × 2 mm column. Detection was achieved by UV-VIS DAD and full scan MS (ESI+, 150–2000 au).

### Combined mass detection by native MS

ESI MS spectra were recorded on an LTQ Orbitrap XL instrument (Thermo Scientific, San Jose, USA) equipped with HESI-II ion source. A samples was injected with an Agilent 1100 μ-autosampler and isocratic pump (Agilent Technologies, Santa Clara, USA). The acquisition was performed using full-scan FTMS mode at 30 K resolution in a positive ions detection mode within 700–2000 mass range. The tube lens voltage was manually tuned to avoid in-source dissociation.

The selectivity of the binding was confirmed by monitoring the mixture of short α-neurotoxin NT II from the *Naja oxiana* venom. Neither HAP nor HAP[L9E] showed combined mass (See supplementary figures S13 and S14).

## Results and Discussion

### Comparison of interactions of αBgt and αCtx with the synthetic peptides by radioligand analysis

**Figure 1:**
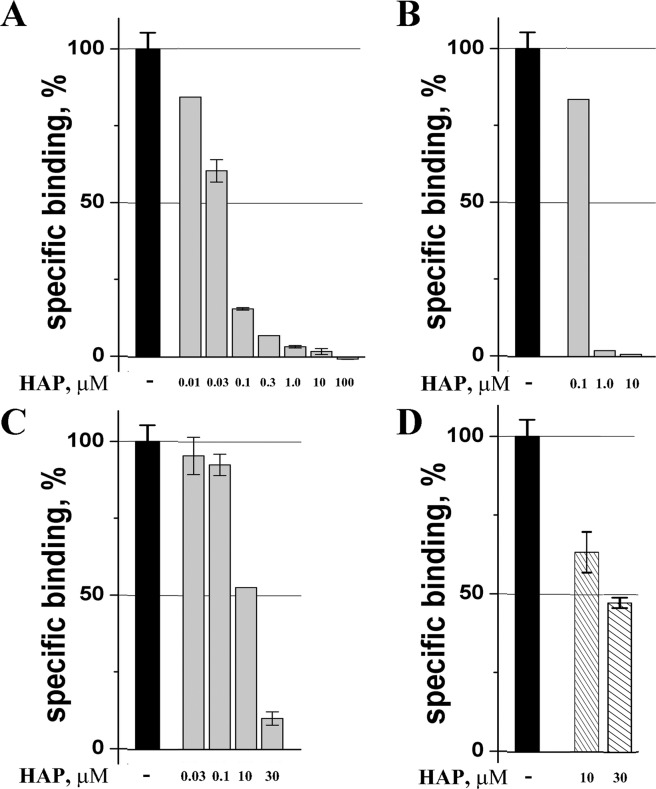
Verification of HAP interaction with αBgt by competitive radioligand analysis. The presence of HAP diminishes dose-dependently the association of ^125^I-αBgt with all tested targets: *Torpedo californica* nAChR (**A**), *Lymnaea stagnalis* AChBP (**B**) and human α7 nAChR (**C**); however, HAP “inhibits” ^125^I-αCtx binding to *T*. *californica* nAChR only partially at concentrations as high as 30 μM (**D**).

Earlier, a high affinity of HAP for αBgt was found in experiments which demonstrated that HAP pre-incubation with biotinylated αBgt diminished binding of the latter to the muscle-type nAChR and the respective concentration dependencies provided the binding parameters. We applied radioligand analysis for the same purpose and compared the HAP effects on the ^125^I-αBgt binding to the muscle-type *Torpedo californica* and human neuronal α7 nAChRs, as well as to Ls-AChBP. Figure 1A–C shows that in all three cases, the presence of the HAP diminished or completely suppressed the ^125^I-αBgt binding to each receptor in a dose-dependent manner. A magnitude of the effect is dependent on the αBgt affinity to the tested targets. Thus, these results confirm earlier publications^18,35^.

In our works on nAChRs, we often used both αBgt and αCtx and considered them as almost identical in terms of inhibitory activity (the advantage of αCtx is its much higher content in the *Naja kaouthia* venom and the simplicity of isolation). Surprisingly, incubation of ^125^I-αCtx even with high concentrations (30 μM) of HAP decreased binding of this radioactive toxin to *T. californica* nAChR only half (Fig. 1D), thus being indicative of a difference in the structural motifs responsible for HAP binding to these two proteins (compare Fig. 1A,D).

HAP showed lower apparent inhibitory activity on *L*. *stagnalis* AChBP (Fig. 1B) and especially on α7 nAChR in GH4C1 cells (Fig. 1C) in radioligand analysis with iodinated α-bungarotoxin. A 300-fold increase in the concentration of HAP is required to get a similar effect with 125I-αCtx. An approximated calculation shows that a “50% inhibition” should be observed on the *L*. *stagnalis* AChBP at 100 μM concentration of HAP, and even at millimolar concentrations in the case of α7 nAChR. Thus, such experiments were not performed.

### Molecular modeling and rational design of HAP analogs

**Figure 2:**
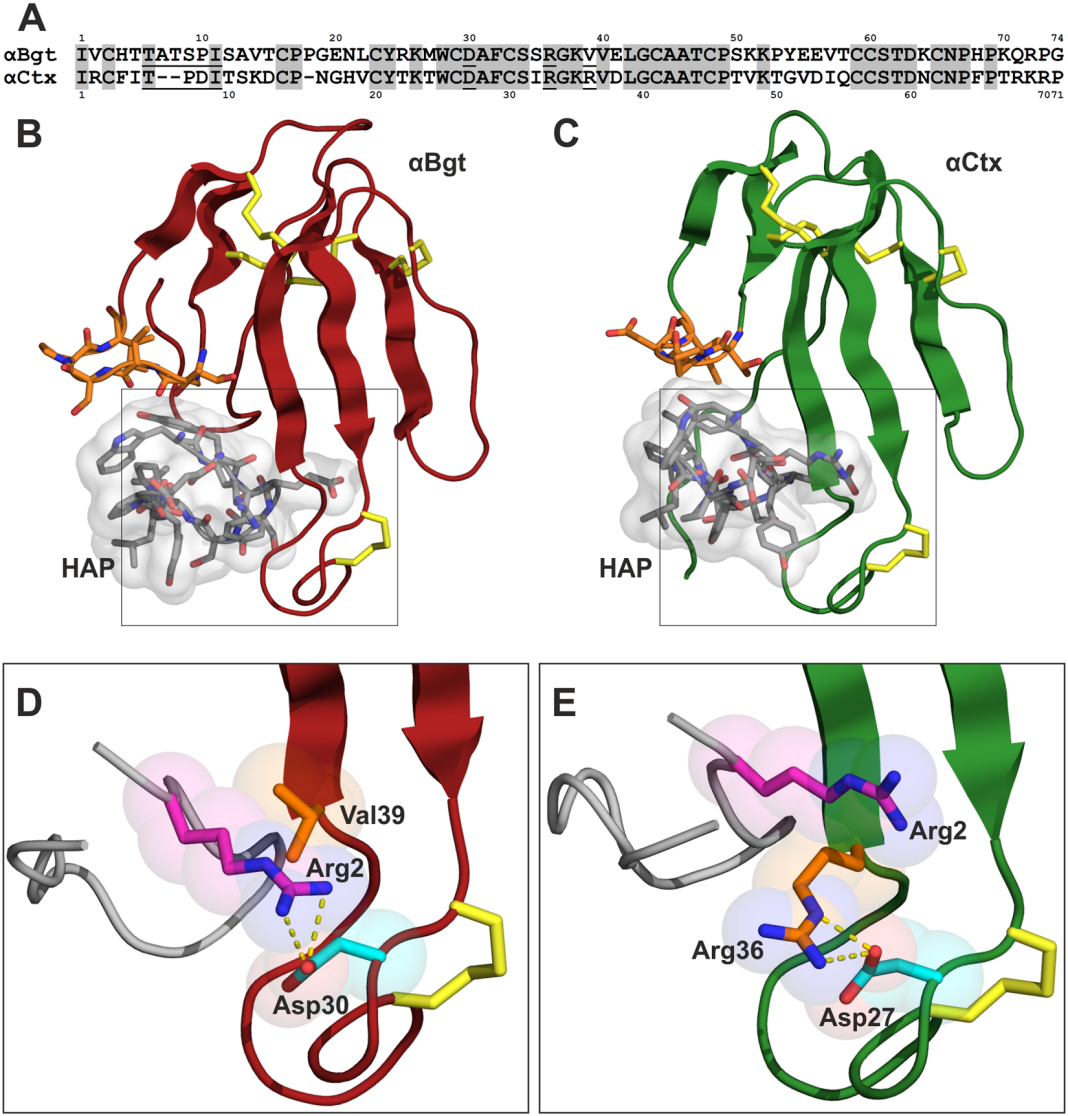
Analysis of the structure of the peptide-toxin complex. (**A**) Amino acid sequence alignment of αBgt and αCtx. The numbering of the residues shown above and under the sequences. The grey background shows identical residues. Residues in the N-terminal and central loops interacting with HAP differentially, according to intermolecular distances analysis, are underlined. (**B**,**C**) complexes of αBgt and αCtx with HAP after 200 ns of MD. αBgt and αCtx are shown by ribbon and coloured in red and green, respectively; HAP is shown by grey sticks and the molecular surface of HAP shown semitransparent; orange sticks show the tip of α-neurotoxins N-terminal loop; yellow sticks show disulfide bridges. (**D**,**E**) magnified fragments inside black boxes on panels (**B**,**C**) respectively. For the clarity, α-neurotoxins are shown partially; HAP is shown as ribbon. Pink sticks show HAP residue Arg2, α-neurotoxins residues Asp30/Asp27 are shown by cyan sticks, orange sticks show Val39/Arg36, nitrogen and oxygen atoms are shown in blue and red, respectively; atoms of the residues are shown as semitransparent spheres; yellow sticks show disulfide bridges; yellow dashed lines show intermolecular contacts. All drawings showing 3D structures were prepared with the PyMOL Molecular Graphics System, version 1.8 (The PyMOL Molecular Graphics System, Version 1.8 Schrödinger, LLC; https://pymol.org/2/).

To explain such a significant difference in HAP binding to αCtx and αBgt, we carried out a computational investigation of these molecular systems. The spatial structure of the αBgt–HAP complex was taken from PDB (1HC9^17^). 3D model of αCtx–HAP complex was built based on the homology with αBgt–HAP complex using αCtx structure derived from its complex with Ls-AChBP (PDB ID: 1YI5^24^). Both complexes were subjected to 200 ns molecular dynamics simulation (MD). Figure 2 shows that HAP may preserve a similar orientation in both complexes. HAP forms multiple hydrophobic and specific intermolecular contacts with αBgt (Supplementary Tables S1–S5), which is in good accordance with the experimental data^17^. However, analysis of contacts along the MD trajectories revealed significant differences in complexes αBgt–HAP and αCtx–HAP due to differences in the neurotoxins structure. In particular, the N-terminal loop of αCtx (residues 6–9) lacks two residues as compared to the analogous loop of αBgt (residues 6–11) (Fig. 2A–C). For that reason, this αCtx fragment forms a single H-bond with HAP, while the analogous loop of αBgt forms five H-bonds (Supplementary Table S1). Besides, Arg2 of HAP forms the H-bond and salt bridge with αBgt residue Asp30, while a bulky side chain of Arg36 forming intramolecular contacts with Asp27 hinders the formation of contact with homologous αCtx residue Asp27 sterically (Fig. 2D,E; Supplementary Tables S1, S2).

**Figure 3:**
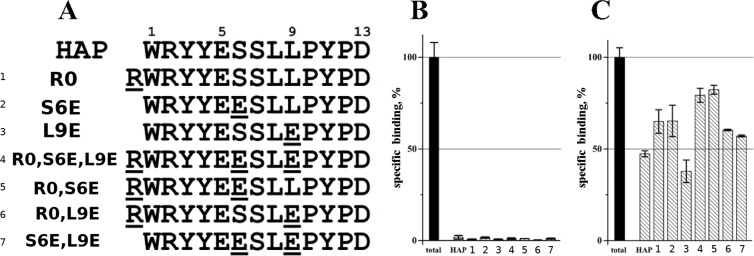
Rational design of the peptides capable to bind of αCtx. (**A**) Amino acid sequence alignment of HAP and its point mutant variants: residues numbering is shown above the sequences. Point mutations are underlined. (**B**) [^125^I]-αBgt specific binding to *T*. *californica* nAChR in the presence of 10 μM HAP and its analogues. (**C**) [^125^I]-αCtx specific binding to *T*. *californica* nAChR in the presence of 30 μM HAP and its analogues. Total binding is measured in the absence of any peptide and expressed in terms of specific binding (% of the general binding minus non-specific binding).

Taking into account experimental data and efforts to design HAP as the best peptide that binds αBgt with high affinity^35^, we tried to follow these studies using a computational approach to design a peptide that would efficiently bind αCtx. The point mutant variants of HAP were proposed in silico based on results of MD simulation of αCtx–HAP and αBgt–HAP as follows. We analyzed intermolecular contacts in the complex αCtx–HAP and taking into account what contacts formed by each residue we tried to replace some HAP residues in a way to obtain complexes where new contacts are structurally (sterically, geometrically) and physical-chemically possible. These complexes with αCtx (as well analogous complexes with αBgt) were subjected to 200 ns MD simulation. Only those peptides, which demonstrated additional intermolecular contacts in the complexes with αCtx and, as far as possible, did not lose contacts in the complexes with αBgt were selected for the following study (Fig. 3A). The designed peptides were synthesized and tested by competitive radioligand analysis (Fig. 3B,C).

**Figure 4:**
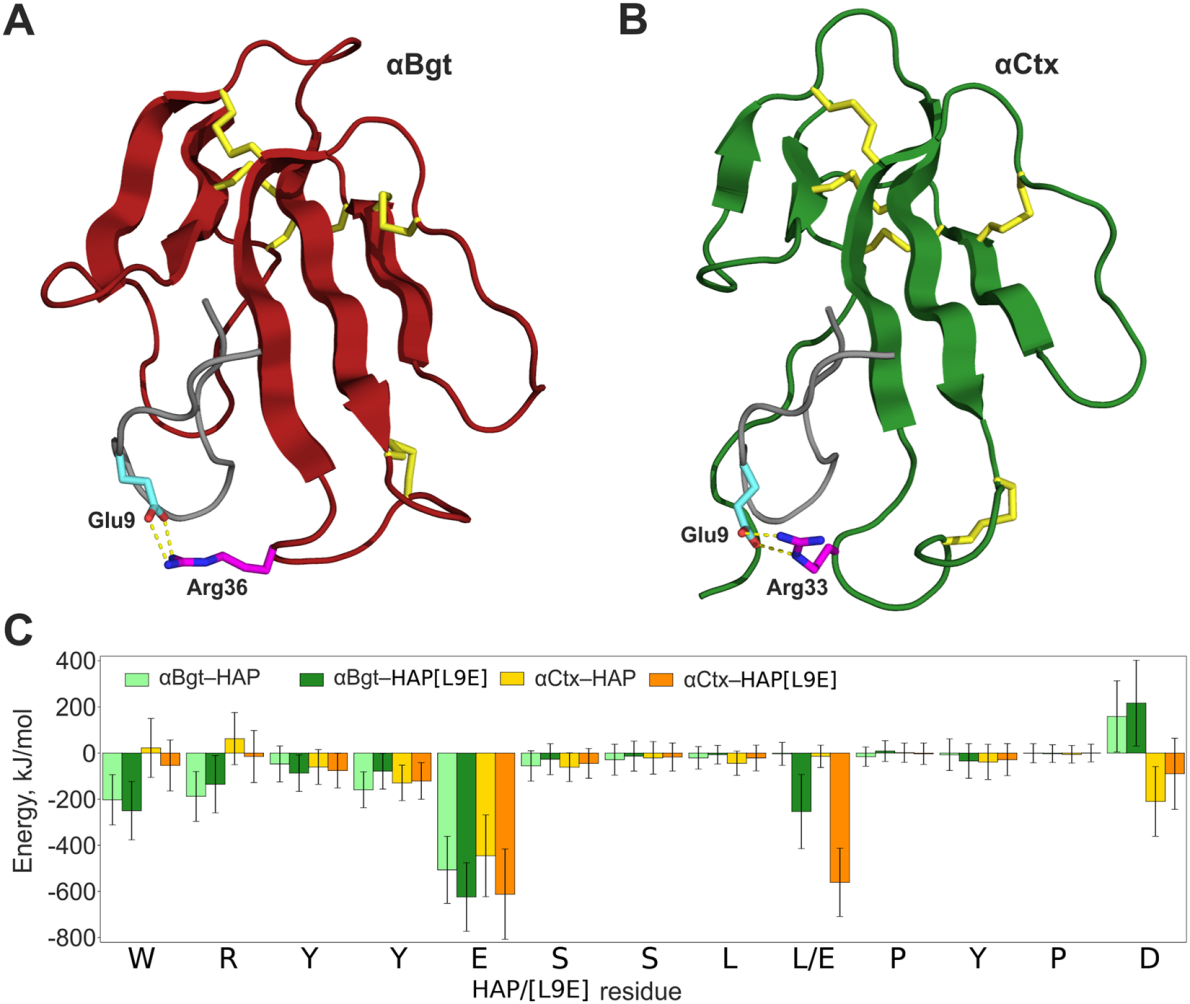
Molecular dynamics analisys of putative complexes between peptide HAP[L9E] (most efficiently binding αCtx) and neurotoxins αCtx and αBgt. (**A**,**B**) complexes of αBgt and αCtx with HAP[L9E] after 200 ns of MD. Polypeptide chains are shown as a ribbon; αBgt coloured in red, αCtx – in green, HAP[L9E] – in grey; yellow sticks show disulfide bridges. Cyan sticks show HAP[L9E] residue Glu9; pink sticks show α-neurotoxins residues Arg36/Arg33; nitrogen and oxygen atoms are shown in blue and red, respectively; yellow dashed lines show intermolecular contacts. (**C**) Interaction energy profiles of HAP/HAP[L9E] in complex with αBgt and αCtx. The bar chart shows amino acid residues’ contributions to the interaction energy averaged over MD simulation. Error bars indicate standard deviations. Glu9 makes a positive impact (negative energy contribution), while Leu9 impact is negligible. All drawings showing 3D structures were prepared with the PyMOL Molecular Graphics System, version 1.8 (The PyMOL Molecular Graphics System, Version 1.8 Schrödinger, LLC; https://pymol.org/2/).

As expected, radioligand analysis confirmed that all designed HAP analogues effectively bind αBgt (Fig. 3B). However, although computational analysis (that during MD suggested for each designed HAP analogue favourable intermolecular contacts with αCtx), only the peptide named HAP[L9E] demonstrated stronger binding of αCtx than did HAP (Fig. 3C). HAP[L9E] peptide with just a single residue change – Leu9Glu – tends to form an additional H-bond and a salt bridge with α-neurotoxins (Fig. 4A,B, Supplementary Tables S1, S2). Notably, the HAP[L9E] complex with αBgt was previously characterized by crystallography^18^, but there was no data in the literature on the HAP[L9E] interaction with αCtx - thus, it is investigated for the first time in this work.

We performed computational analysis of complex-bound HAP[L9E]/HAP amino acid residues’ contributions to the interaction energy. The resulting interaction energy profile (Fig. 4C) demonstrates a significant negative energy contribution of HAP[L9E] residue Glu9, while the contribution of analogous HAP residue Leu9 is negligible. This result assumes that HAP[L9E] is to bind αCtx with considerably higher affinity than HAP does.

The results of the computational analysis suggest that peptide HAP[L9E] binds to αCtx substantially more efficiently then HAP. Interestingly, most of nAChR α-subunits C-loops also contain Glu or Asp residue in the same positions. Thus, HAP[L9E] comprises a more accurate model of the receptors binding site than the original HAP. To evaluate HAP[L9E] binding affinity to αCtx, we applied a label-free approach for measuring binding constants in HAP-toxin pairs based on intrinsic protein fluorescence spectroscopy.

### A label-free fluorescence method to detect binding of HAP and its analogs to three-finger α-neurotoxins

It should be noted that such indirect methods as monitoring of radio- or fluorescent-labelled toxin binding to the nAChR or GABAAR do not allow to measure constants of dissociation of studied peptides. However, these parameters are essential to rationally interpret molecular modeling and to design new HAP analogs that would be more universal at binding not only αBgt but also its such homologs as αCtx. That is why we applied an experimental approach based on the intrinsic protein fluorescence to assess binding of HAP (and its analogues) to αCtx and αBgt.

Intrinsic proteins fluorescence at UV excitation is due to aromatic amino acid residues in its structure, and Trp is the major fluorophore because of its higher fluorescence quantum yield. Trp fluorescence emission properties such as intensity, fluorescence lifetime and spectral band shape are sensitive to their local microenvironment and are commonly used to study proteins’ interaction. Namely, Trp fluorescence sensitivity to Trp residue mobility during the excited state lifetime, its exposure to the solvent and the polarity of the local environment allow investigation of protein conformational changes, including those caused by complex formation^36^.

**Figure 5:**
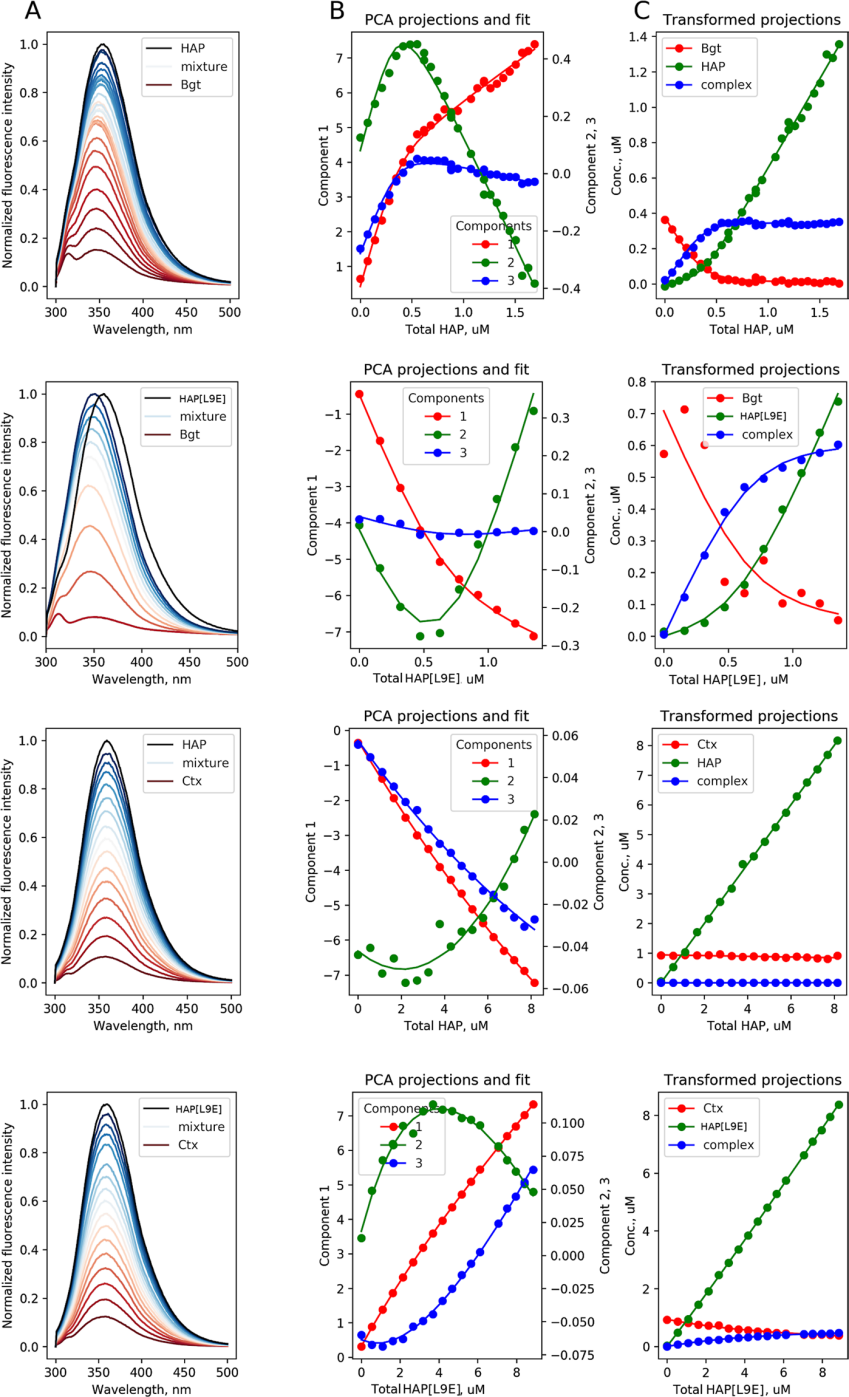
Fluorescence spectra and fitting results for αBgt-HAP, αBgt-HAP[L9E], αCtx-HAP, αCtx-HAP[L9E] toxin-peptide pairs. (**A**) Normalized fluorescence spectra of toxin (dark red), peptide (black) and their mixtures with different concentration ratios (dark red to blue). (**B**) Spectra projections on the first three principal components (dots) and the fit obtained using 1:1 complexation model (solid lines). The nonlinear dependence of the first projection on peptide concentration demonstrates complexation in systems, and the 1:1 model is in a good agreement with the projections obtained from the experimental data. (**C**) Dependence of partial concentrations of peptide, toxin and complex on total peptide concentrations obtained from PCA projections.

A blue shift of spectra was observed upon addition of peptides to αBgt, and the dependence of fluorescence spectra maxima on peptides concentration was nonmonotonous, being indicative of binding in the system (Fig. 5). In contrast to this, the dependences of the spectra maxima position on peptides concentration for αCtx were monotonous, integral fluorescence intensity dependence on peptide concentration was closer to linear, and the observed fluorescence spectra (see Fig. 5A) demonstrated only slight band shape variation. Hence, the application of the simple fitting procedure of experimental data for a single wavelength or integral intensity was complicated. So, we used the spectra processing algorithm based on the composition of principal component analysis (PCA) and nonlinear least square (NLS) fitting described in detail elsewhere^37^ and given as a summary with examples in Supporting information section 2. Application of PCA allowed us to make the interaction between the peptide and toxin and the arising complex formation more evident.

As can be seen from Fig. 5B, the PCA projections obtained for the system of toxin (αBgt) and peptide (HAP), interacting with high affinity, demonstrate a nonlinear dependence on the toxin concentration, thus providing evidence for complex formation. The dependences of the first three projections obtained from PCA on the total peptide concentration were further fitted with a 1:1 complex formation model (K_d_ = [α-neurotoxin][binding peptide]/[Complex]) providing the value of dissociation constant (see in Table 1).

**Table 1:** Dissociation constants for peptide-toxin pairs obtained from PCA+NLS fitting of fluorescence spectra.

Toxin	Peptide	K_d_, nM
αBgt	HAP	29 ± 8
αBgt	HAP[L9E]	91 ± 33
αCtx	HAP	>10,000
αCtx	HAP[L9E]	7000 ± 1000

The applied algorithm allows transforming the projections vector into the concentration vector using the rotation matrix (**P** in the notion of^37^) calculated using the obtained affinity constant values. Hence, one can plot concentration dependence for free peptide, toxin and their complex on the total peptide concentration (see Fig. 5C). Noteworthy, αBgt demonstrated a higher affinity for both HAP and HAP[L9E] as compared to αCtx, however, while no interaction was observed for the HAP-αCtx system, it was clearly shown that the HAP[L9E] peptide binds to αCtx (see Fig. 5C and Table 1).

Both neurotoxins αCtx and αBgt, as well as both peptides HAP and HAP[L9E], each, contain one tryptophan residue in their structure (Figs. 2A and 3A). Notably, each tryptophan residue is involved in multiple intermolecular contacts formation (Supplementary Tables S1–S5). To ascertain the fluorescence spectrum changes by the complex formation, we conducted a computational investigation of the molecular surface of each tryptophan residue in the complexes and free αCtx, αBgt, HAP and HAP[L9E]. For this purpose, we conducted an MD simulation of these polypeptide molecules in an explicitly defined water environment.

**Figure 6:**
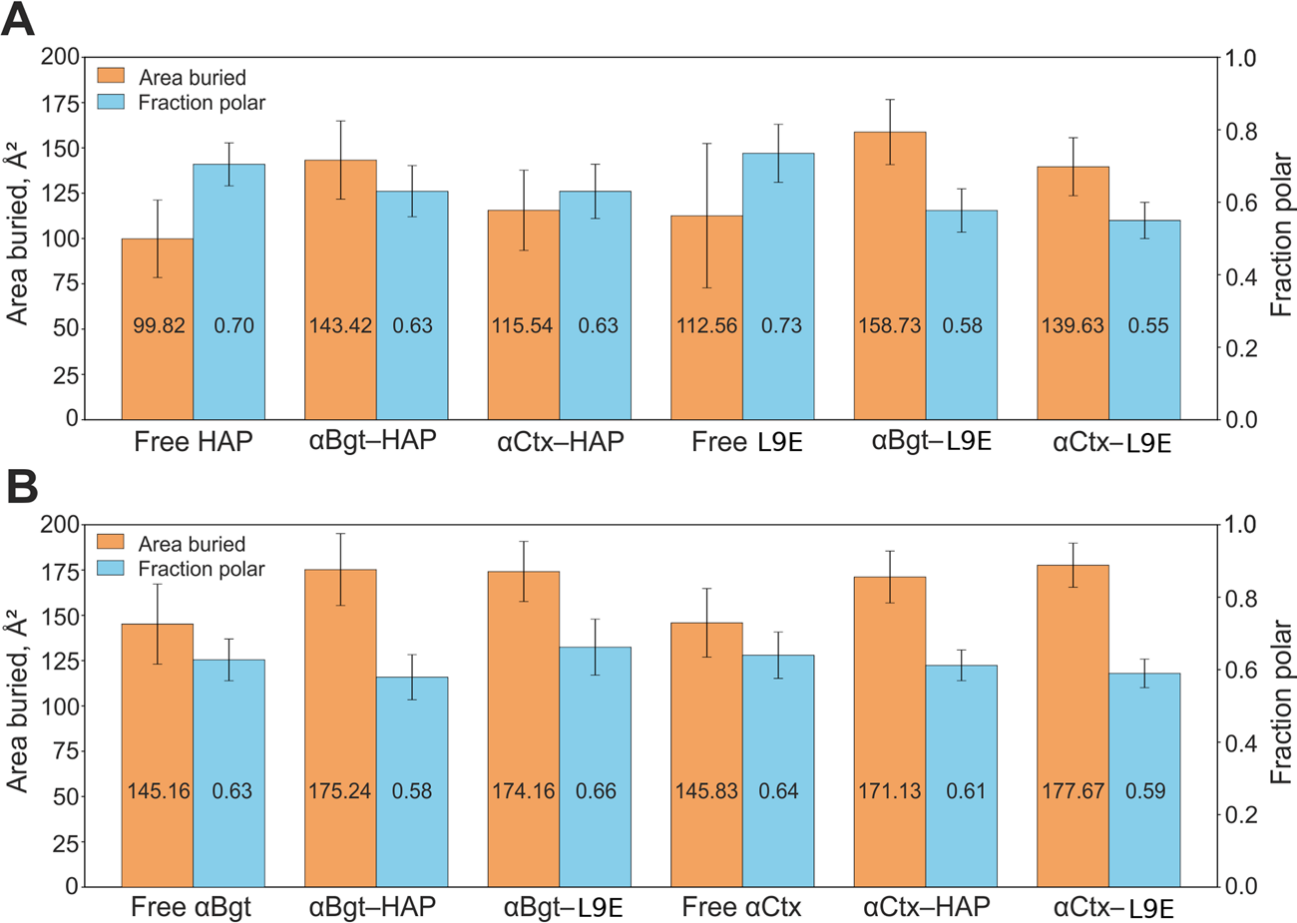
Area buried and fraction polar averaged over MD simulation for (**A**) Trp1 in free and complex-bound HAP/HAP[L9E] and (**B**) Trp28/Trp25 in free and complex-bound αBgt/αCtx. Error bars indicate standard deviations.

It was shown that the buried surface area (“area buried”, Ab^38^) of HAP/HAP[L9E] Trp1 residue side chain in the complexes with αBgt is ~40/45 Å^2^ greater than in free peptides, and in the complexes with αCtx it is greater for ~15/39 Å^2^ (Fig. 6A). The fraction of the side chain area that is exposed to polar atoms (“fraction polar”, Fp^38^) of HAP/HAP[L9E] Trp1 residue in the complexes with αBgt is ~7/15% smaller than in free peptides, and in the complexes with αCtx it is smaller by ~7/18%. On the other hand, Ab of αBgt/αCtx Trp28/Trp25 residue side chain in the complexes with HAP is ~30/26 Å^2^ greater than in free α-neurotoxins, and in the complexes with HAP[L9E] it is greater for ~29/32 Å^2^ (Fig. 6B). Nevertheless, Fp of αBgt/αCtx Trp28/Trp25 residues in the complexes do not differ dramatically from Fp value in free peptides: it is for ~5/3% smaller in the complexes with HAP, and for ~3/5% smaller in the complexes with HAP[L9E].

These results show that in HAP as well as in HAP[L9E], the Trp1 side chain becomes more buried in the complexes with the α-neurotoxins, while the polarity of its molecular environment decreases. This observation is consistent with the results of fluorescence analysis: in case of strong Trp microenvironment influence by complexation process, the peak of the tryptophan fluorescence spectrum shifts to the shortwave region, and the dependence of spectra maxima position on peptide concentration at constant toxin concentration becomes nonmonotonous. The most pronounced variations of spectra and nonmonotonous dependence of spectra maxima correspond to αBgt-HAP and αBgt-HAP[L9E] complexes, which are characterized by the most significant changes of Ab parameter of Trp1 of the peptide (see Fig. 6A). For αCtx-HAP and αCtx-HAP[L9E] pairs, the variations of spectra due to complexation are less pronounced, with Trp1 Ab parameter changes due to complexation being smaller (see Fig. 6A). Vice versa, although the side chain of Trp28/Trp25 in αBgt/αCtx becomes more buried in the complexes with the peptides, it would hardly affect the fluorescence spectrum, since the polarity of Trp28/Trp25 molecular environment does not decrease much.

### Validation of binding constants by gel-filtration chromatography and mass-spectrometry

Using the steady-state fluorescence measurement followed by PCA, we have detected the interaction of HAP with αCtx. However, we found it necessary to validate the results of the label-free fluorescence spectroscopy by comparing them to well-established methods of direct ligand-receptor interaction measurement. To confirm that observed effects are due to the specific interaction of HAP or HAP[L9E] with α-neurotoxins, we used gel-filtration.

**Figure 7:**
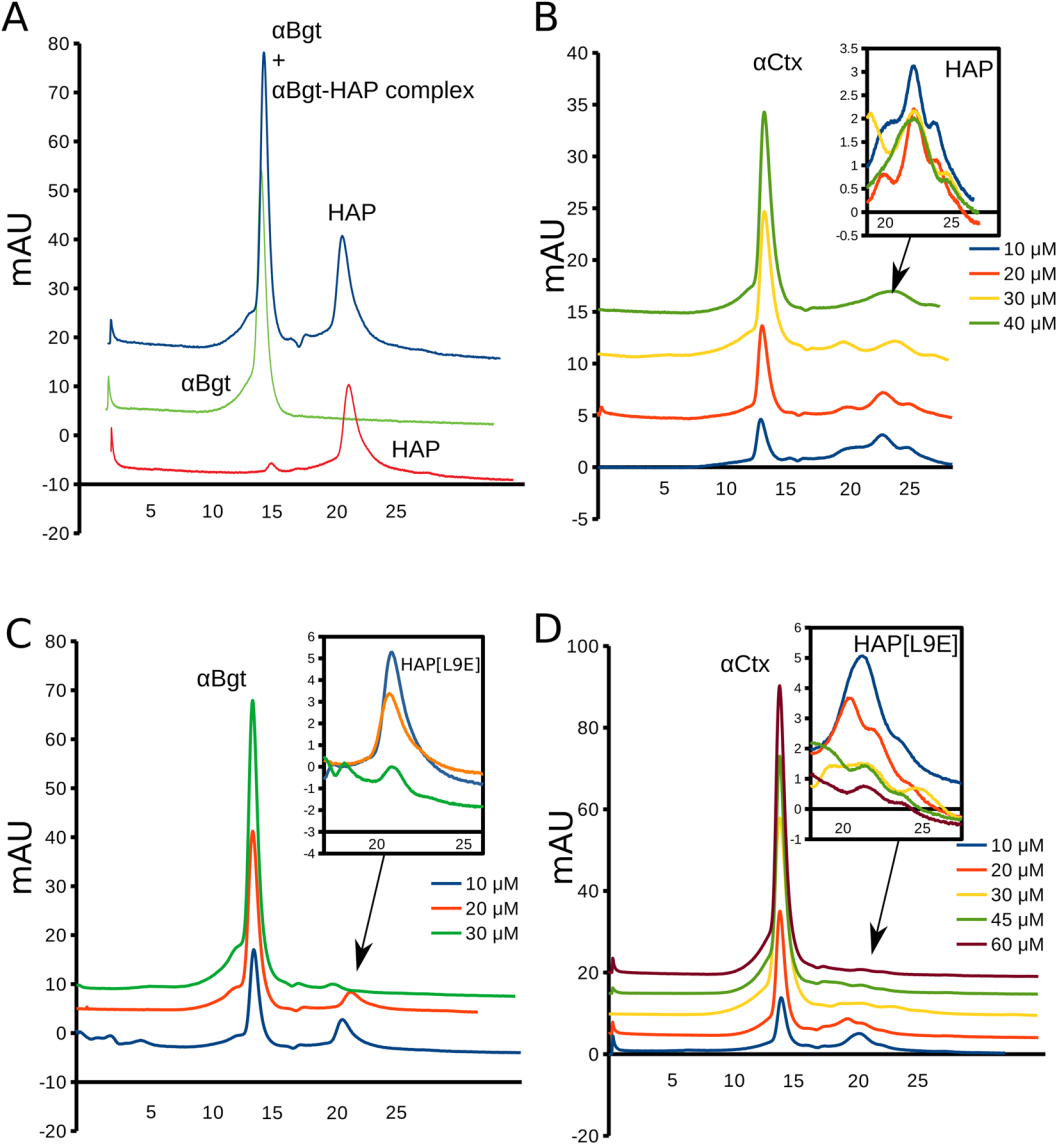
Monitoring the peptide-toxin complex formation *via* gel-filtration. (**A**) Gel-filtration profile of αBgt (green), HAP (orange) and their mixture (blue) on the Superdex 75 10/300 GL column. Pure HAP and αBgt are well separated on this column, thus allowing to monitor the formation of the complex between HAP and three-finger toxin (see Fig. S10 for the LC-MS). (**B**) No signs of HAP-αCtx complex formation observed on the gel-filtration profile of their mixture. (**C**) HAP[L9E] analogue of HAP interacts with αBgt in a concentration-dependent manner as can be monitored via reduction of the respective peak on gel-filtration profile with increasing concentrations of αBgt. (**D**) HAP[L9E] analogue of HAP also interacts with αCtx in a concentration-dependent manner and reduction of its peak can be monitored on the gel-filtration profile with increasing concentrations of αCtx.

Gel-filtration on the column Superdex 75 10/300 GL is capable of separating molecules in the mass range 3000–70000 Da. HAP mass is 1689, αBgt and αCtx masses are about 7800 Da. Thus, 1:1 complex of HAP or its analogue with αBgt or αCtx should be around 9500 Da. Indeed, gel-filtration on Superdex 75 10/300 GL shows a sufficient retention time difference between HAP and αBgt (Fig. 7A). However, we expected that gel-filtration would not adequately resolve HAP-αBgt complex from un-bound αBgt. That is why we deployed the LC-MS analysis of the first peak. Supplementary Fig. S10 shows the data from LC-MS of the first peak on HAP-αBgt mixture chromatogram (blue line in Fig. 7A). Both HAP and αBgt molecules detected in the first peak, showing that HAP which is significantly smaller co-elutes with αBgt during gel filtration, confirming strong binding of HAP to αBgt.

Figure 7B shows the gel-filtration of the series mixtures of HAP with αCtx with increasing concentrations of the later. The second peak corresponding to the free HAP does not reduce upon αCtx concentration increase, suggesting that complex between HAP and αCtx is so weak that it does not survive the gel-filtration conditions. Modified peptide HAP[L9E], on the contrary, shows strong complexation with both αBgt (Fig. 7C) and αCtx (Fig. 7D). We performed several co-elutions for each pair: HAP[L9E]-αBgt and HAP[L9E]-αCtx using increasing concentrations of the particular toxins. Higher concentrations of the toxins bind more considerable amount of the HAP[L9E] peptide which leads to a gradual decrease of the second peak on the gel-filtration elution profile because higher quantities of the peptide elute in the first peaks. Therefore, HAP[L9E] peptide, which exhibited binding to both αCtx and αBgt in fluorescence measurements, showed a concentration-dependent interaction (monitored as free HAP[L9E] peak area reduction) with both αBgt (Fig. 7C) and αCtx (Fig. 7D), thus confirming our observations from PCA of Trp fluorescence spectra and molecular modeling. LC-MS confirmed co-elution of HAP[L9E] with both αBgt and αCtx in the first peaks (Figs. S11–S12).

**Figure 8:**
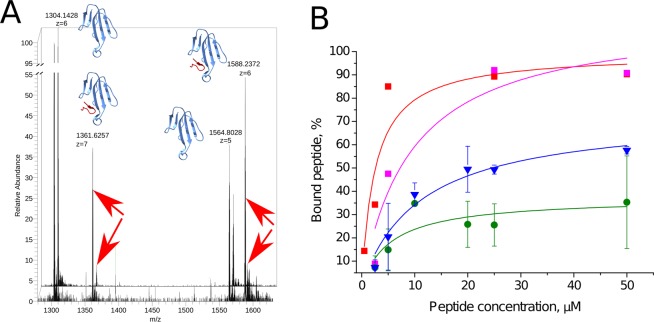
ESI-MS observation of the toxin and binding peptide combined mass. (**A**) superimposed spectra of 1:1 αCtx to HAP[L9E] mixture (background spectrum) and 1:10 αCtx to HAP[L9E] mixture (foreground spectrum). Note that m/z peaks (pointed by red arrows) indicating the combined αCtx + HAP[L9E] mass are higher in the 1:10 than in 1:1 mixture. Titration of αCtx/αBgt with HAP/HAP[L9E] and monitoring the intensities of the respective peaks allows the binding evaluation. (**B**) Titration curves fitted by the one-site binding equation: αBgt and HAP (red), αBgt and HAP[L9E] (magenta), αCtx and HAP[L9E] (blue), αCtx and HAP (green). Even though both αCtx curves do not saturate at 100% (in contrast to αBgt), complex HAP[L9E] saturates at a higher level (73 + 3%) than HAP (37 + 8%), indicating stronger binding between the toxin and mutated peptide. Drawings showing 3D structures were prepared with the UCSF Chimera, version 1.12 (http://www.rbvi.ucsf.edu/chimera/).

Co-elution in the gel-filtratinon confirms that HAP[L9E] binds the αCtx. But how strong and how specific this interaction might be? To investigate the interaction in more detail we performed electrospray ionisation mass-spectrometry (ESI-MS) with Orbitrap detection of the non-covalent complexes of toxins and binding peptides (Fig. 8). The principles of this method was described by Tjernberg *et al*.^39^.

Indeed, we observe the combined m/z on the HAP[L9E] and αCtx mixture spectra (Fig. 8A). And its intensity depends on the HAP[L9E] concentration, complementing the gel-filtration experiment. Titration of αCtx/αBgt with HAP/HAP[L9E] and monitoring the intensities of the respective peaks followed by fitting the dependency to one-site competition equation (Fig. 8B) allowed us to prove the complexation of HAP[L9E] with αCtx directly. We also detected αCtx complexation with HAP, which confirms the radioligand binding data (Fig. 3C). It should be noted that αCtx seems to be not fully saturated neither by HAP nor HAP[L9E] which did not allow us to estimate binding constants. This observation confirms that the complexes with αCtx are weaker than the respective complexes with αBgt and partially degrade during electrospray ionisation. However, HAP[L9E] curve shows a higher saturation level which confirms that the αCtx-HAP[L9E] complex is more stable than αCtx-HAP.

## Conclusions

Here, using competitive radioligand analysis, intrinsic protein fluorescence spectroscopy with PCA-based processing, gel-filtration and mass-spectrometry, we confirmed the earlier finding of Harel *et al*. that a 13-membered peptide HAP has an extremely high affinity for αBgt. Unexpectedly, we detected only weak binding of HAP to αCtx. Thus, αBgt and αCtx, known to be very similar in their affinity for nAChRs, differ in this characteristic towards AChBPs, GABAA receptors and, as shown here, to such C-loop analogue as HAP. With molecular dynamics, we deduced the molecular properties of the affinity peptides complexes and suggested the way to improve affinity toward αCtx. Using radioligand competition method, we selected the most effective αCtx binding peptide. To measure the binding constants label-free, we acquired intrinsic Trp fluorescence measurement with spectra principal component analysis. Information found in this article could be used in the design of experiments involving HAP-based tags and fluorescent derivatives of αBgt and αCtx.

## Supplementary information

Supplementary Information.
