# Supplementary material for: Complex approach for analysis of snake venom α-neurotoxins binding to HAP, the high-affinity peptide
Source: Sci Rep. 2020 Mar 2;10:3861. doi: 10.1038/s41598-020-60768-y (PMC7052197; doi:10.1038/s41598-020-60768-y)
Supplement: Supplementary file 1 — Supplementary Information. [file 41598_2020_60768_MOESM1_ESM.pdf]

Complex approach for analysis of snake venom α-neurotoxins binding to HAP, the high-affinity peptide

Denis S. Kudryavtsev*[1], Valentin M. Tabakmakher*[1,2], Gleb S. Budylin*[3], Natalia S. Egorova[1], Roman G. Efremov[1,4,5], Igor A. Ivanov[1], Svetlana Yu. Belukhina[6], Artem V. Egorov[1], Igor E. Kasheverov[1,7], Elena V. Kryukova[1], Irina V. Shelukhina[1], Evgeny A. Shirshin[8,9], Nadezhda G. Zhdanova[8], Maxim N. Zhmak[1] and Victor I. Tsetlin‡[1,10]

*-authors contributed equally

[1]Shemyakin-Ovchinnikov Institute of Bioorganic Chemistry, Russian Academy of Sciences, Moscow, 117997, Russia

[2]School of Biomedicine, Far Eastern Federal University, Vladivostok, 690950, Russia

[3]Faculty of Physics, National Research University Higher School of Economics, Moscow, 101000, Russia

[4]National Research University Higher School of Economics, Moscow, 101000, Russia

[5]Moscow Institute of Physics and Technology (State University), Dolgoprudny, 141701, Moscow Oblast, Russia

[6]Faculty of Biotechnology, Moscow State University, Moscow, 119991, Russia

[7]Sechenov First Moscow State Medical University, Institute of Molecular Medicine, Moscow, 119991, Russia

[8]Department of Physics, M.V. Lomonosov Moscow State University, Moscow, 119992, Russia

[9]Institute of spectroscopy of the Russian Academy of Sciences, Troitsk, Moscow, 108840, Russia

[10]PhysBio of MePhi, Kashirskoe Ave. 31, 115409 Moscow, Russia;

‡**Correspondence to: vits@ibch.ru**

Supporting information
Section 1

Supplementary Table S1.

H-bonds observed during 200ns MD of αBgt-HAP, αCtx-HAP, αBgt-HAP[L9E], and αCtx-HAP[L9E].

Hydrogen bonds*											
αBgt-HAP			αCtx-HAP			αBgt-HAP[L9E]			αCtx-HAP[L9E]		
**HAP residue**	**αBgt residue**	**lifetime ****	**HAP residue**	**αCtx residue**	**lifetime**	**HAP[L9E] residue**	**αBgt residue**	**lifetime**	**HAP[L9E] residue**	**αCtx residue**	**lifetime**
TRP1	THR6	m				TRP1	THR6	m			
TRP1	ALA7	m				TRP1	ALA7	m			
						TRP1	THR8	m			
TRP1	SER9	m				TRP1	SER9	m			
			TRP1	ASP38	m	TRP1	GLU41	m	TRP1	ASP38	m
ARG2	ASP30	m									
						ARG2	GLU41	m			
						TYR3	ALA7	m			
TYR3	THR8	s				TYR3	THR8	m			
TYR3	SER9	m				TYR3	SER9	m			
			TYR3	ASP8	l				TYR3	ASP8	l
TYR3	ILE11	s									
TYR3	VAL40	l	TYR3	VAL37	l	TYR3	VAL40	l	TYR3	VAL37	l
						TYR3	HIS68	s			
TYR3	GLN71	s									
TYR3	GLY74	s									
TYR4	ASP30	l	TYR4	ASP27	l						
TYR4	ARG36	m	TYR4	ARG33	m						
			TYR4	ARG36	m				TYR4	ARG36	l
						TYR4	GLY37	m			
						TYR4	LYS38	m			
TYR4	HIS68	m				TYR4	HIS68	m			
						GLU5	SER35	m			
						GLU5	ARG36	m			
GLU5	LYS38	l	GLU5	LYS35	l	GLU5	LYS38	l	GLU5	LYS35	l
			GLU5	THR67	m	GLU5	LYS70	s	GLU5	THR67	m
			GLU5	ARG68	s						
SER6	ARG36	m	SER6	ARG33	l				SER6	ARG33	m
SER6	HIS68	s									
SER6	LYS70	s	SER6	THR67	s	SER6	LYS70	s	SER6	THR67	s
			SER6	ARG68	m						
									SER6	LYS69	m
			SER6	ARG70	m				SER6	ARG70	l
SER7	ARG36	m	SER7	ARG33	s				SER7	ARG33	m
SER7	HIS68	m									
			SER7	ARG68	s				SER7	ARG68	s
			SER7	ARG70	m				SER7	ARG70	s

	SER7 PRO71 s		
LEU8 GLN71 s	LEU8 ARG68 l		LEU8 THR67 m<br>LEU8 ARG68 m
		GLU9 ARG36 m	GLU9 ARG33 m
	TYR11 PRO71 m	TYR11 ASP30 m<br>TYR11 ARG36 m	
ASP13 SER9 s	ASP13 ARG36 s		

* All the contacts in the table "aligned" considering αBgt-αCtx and HAP-HAP[L9E] amino acid sequence alignment (Fig 2A, 3A). Each contact in the αBgt-HAP complex is put in correspondence with analogous contacts in the complexes αCtx-HAP, αBgt-HAP[L9E], and αCtx-HAP[L9E]. In case when an analogous contact was not observed during MD, the corresponding cell in the table is empty.

** Lifetime of each contact counted as part of MD-trajectory total time (200ns). "s" stands for short-living contacts: lifetime is less than 10 %; "m" stands for medium-living contacts: lifetime is less than 50 %; "l" – long stands for long-living contacts: lifetime is greater than 50 %.

Supplementary Table S2.

Salt bridges observed during 200ns MD of αBgt-HAP, αCtx-HAP, αBgt-HAP[L9E], and αCtx-HAP[L9E].

**Salt bridges***											
**αBgt-HAP**			**αCtx-HAP**			**αBgt-HAP[L9E]**			**αCtx-HAP[L9E]**		
**HAP residue**	**αBgt residue**	**lifetime**	**HAP residue**	**αCtx residue**	**lifetime**	**HAP[L9E] residue**	**αBgt residue**	**lifetime**	**HAP[L9E] residue**	**αCtx residue**	**lifetime**
ARG2	ASP30	m				ARG2	GLU41	m			
GLU5	LYS38	m	GLU5	LYS35	m	GLU5	LYS38	s	GLU5	LYS35	m
						GLU9	ARG36	m	GLU9	ARG33	m
			ASP13	ARG36	m						

* All the contacts in the table “aligned” considering αBgt-αCtx and HAP-HAP[L9E] amino acid sequence alignment (Fig 2A, 3A). Each contact in the αBgt-HAP complex is put in correspondence with analogous contacts in the complexes αCtx-HAP, αBgt-HAP[L9E], and αCtx-HAP[L9E]. In case when an analogous contact was not observed during MD, the corresponding cell in the table is empty.

** Lifetime of each contact counted as part of MD-trajectory total time (200ns). “s” stands for short-living contacts: lifetime is less than 10 %; “m” stands for medium-living contacts: lifetime is less than 50 %; “l” – long stands for long-living contacts: lifetime is greater than 50 %.

Supplementary Table S3.

Stacking and π-π interactions observed during 200ns MD of αBgt-HAP, αCtx-HAP, αBgt-HAP[L9E], and αCtx-HAP[L9E].

**Stacking / π-π interactions***											
**αBgt-HAP**			**αCtx-HAP**			**αBgt-HAP[L9E]**			**αCtx-HAP[L9E]**		
**HAP residue**	**αBgt residue**	**lifetime**	**HAP residue**	**αCtx residue**	**lifetime**	**HAP[L9E] residue**	**αBgt residue**	**lifetime**	**HAP[L9E] residue**	**αCtx residue**	**lifetime**
ARG2	TRP28	m	ARG2<br>ARG2	TRP25<br>ARG36	m<br>m	ARG2	TRP28	l	ARG2	TRP25	l
TYR4	ARG36	m	TYR4<br>TYR4<br>TYR4	ARG33<br>PHE29<br>ARG36	m<br>m<br>m	TYR4	ARG36	m	TYR4<br>TYR4<br>TYR4	ARG33<br>PHE29<br>ARG36	m<br>s<br>s
TYR11	ARG36	l	TYR11<br>TYR11	ARG33<br>ARG36	l<br>m				TYR11	ARG36	l

* All the contacts in the table "aligned" considering αBgt-αCtx and HAP-HAP[L9E] amino acid sequence alignment (Fig 2A, 3A). Each contact in the αBgt-HAP complex is put in correspondence with analogous contacts in the complexes αCtx-HAP, αBgt-HAP[L9E], and αCtx-HAP[L9E]. In case when an analogous contact was not observed during MD, the corresponding cell in the table is empty.

** Lifetime of each contact counted as part of MD-trajectory total time (200ns). "s" stands for short-living contacts: lifetime is less than 10 %; "m" stands for medium-living contacts: lifetime is less than 50 %; "l" – long stands for long-living contacts: lifetime is greater than 50 %.

Supplementary Table S4.

Cation-π interactions observed during 200ns MD of αBgt-HAP, αCtx-HAP, αBgt-HAP[L9E], and αCtx-HAP[L9E].

Cation-π interactions*											
**αBgt-HAP**			**αCtx-HAP**			**αBgt-HAP[L9E]**			**αCtx-HAP[L9E]**		
**HAP residue**	**αBgt residue**	**Lifetime****	**HAP residue**	**αCtx residue**	**lifetime**	**HAP[L9E] residue**	**αBgt residue**	**lifetime**	**HAP[L9E] residue**	**αCtx residue**	**lifetime**
ARG2	TRP28	l	ARG2	TRP25	m	ARG2	TRP28	l	ARG2	TRP25	l
ARG2	PHE32	s	ARG2	PHE29	m						
TYR4	ARG36	l	TYR4	ARG33	l	TYR4	ARG36	l	TYR4	ARG33	l
			TYR4	ARG36	m				TYR4	ARG36	l
TYR11	ARG36	l	TYR11	ARG33	l	TYR11	ARG36	m			
			TYR11	ARG36	m				TYR11	ARG36	l

* All the contacts in the table “aligned” considering αBgt-αCtx and HAP-HAP[L9E] amino acid sequence alignment (Fig 2A, 3A). Each contact in the αBgt-HAP complex is put in correspondence with analogous contacts in the complexes αCtx-HAP, αBgt-HAP[L9E], and αCtx-HAP[L9E]. In case when an analogous contact was not observed during MD, the corresponding cell in the table is empty.

** Lifetime of each contact counted as part of MD-trajectory total time (200ns). “s” stands for short-living contacts: lifetime is less than 10 %; “m” stands for medium-living contacts: lifetime is less than 50 %; “l” – long stands for long-living contacts: lifetime is greater than 50 %.

Supplementary Table S5.
Hydrophobic contacts observed during 200ns MD of αBgt-HAP, αCtx-HAP, αBgt-HAP[L9E], and αCtx-HAP[L9E].

**Hydrophobic contacts***											
**αBgt-HAP**			**αCtx-HAP**			**αBgt-HAP[L9E]**			**αCtx-HAP[L9E]**		
**HAP residue**	**αBgt residue**	**lifetime****	**HAP residue**	**αCtx residue**	**lifetime**	**HAP[L9E] residue**	**αBgt residue**	**lifetime**	**HAP[L9E] residue**	**αCtx residue**	**lifetime**
TRP1	THR5	m	TRP1	ILE5	m	TRP1	THR5	l	TRP1	ILE5	m
TRP1	THR6	l	TRP1	THR6	l	TRP1	THR6	l	TRP1	THR6	l
TRP1	ALA7	l				TRP1	ALA7	l			
TRP1	THR8	l				TRP1	THR8	l			
TRP1	SER9	l	TRP1	PRO7	l	TRP1	SER9	l	TRP1	PRO7	l
TRP1	PRO10	l	TRP1	ASP8	l	TRP1	PRO10	l	TRP1	ASP8	l
TRP1	ILE11	l	TRP1	ILE9	l	TRP1	ILE11	l	TRP1	ILE9	l
TRP1	SER12	m									
			TRP1	TYR21	m	TRP1	TYR24	l	TRP1	TYR21	m
			TRP1	LYS23	m				TRP1	LYS23	m
TRP1	TRP28	m	TRP1	TRP25	m	TRP1	TRP28	l	TRP1	TRP25	l
TRP1	VAL39	m	TRP1	ARG36	l						
									TRP1	VAL37	m
TRP1	GLU41	l	TRP1	ASP38	l	TRP1	GLU41	l	TRP1	ASP38	l
TRP1	LEU42	m	TRP1	LEU39	l	TRP1	LEU42	l	TRP1	LEU39	l
						TRP1	GLY43	l			
TRP1	PRO67	m									
									TRP1	PRO66	m
TRP1	GLN71	m				TRP1	GLN71	m			
TRP1	PRO73	m									
ARG2	THR6	l	ARG2	THR6	l	ARG2	THR6	l	ARG2	THR6	l
ARG2	ALA7	l				ARG2	ALA7	l			
ARG2	THR8	m				ARG2	THR8	l			
ARG2	SER9	m	ARG2	PRO7	l	ARG2	ILE11	m	ARG2	PRO7	l
ARG2	ILE11	m	ARG2	ILE9	m				ARG2	ILE9	l
			ARG2	TYR21	m	ARG2	TYR24	l	ARG2	TYR21	m
ARG2	LYS26	m	ARG2	LYS23	m	ARG2	LYS26	l	ARG2	LYS23	l
			ARG2	THR24	m	ARG2	MET27	l	ARG2	THR24	l
ARG2	TRP28	l	ARG2	TRP25	l	ARG2	TRP28	l	ARG2	TRP25	l
			ARG2	CYS26	m	ARG2	CYS29	l	ARG2	CYS26	l
ARG2	ASP30	l				ARG2	ASP30	l			
ARG2	ALA31	l				ARG2	ALA31	m			
ARG2	PHE32	l	ARG2	PHE29	l				ARG2	PHE29	m
ARG2	ARG36	l	ARG2	ARG33	l						
						ARG2	LYS38	m	ARG2	LYS35	m
ARG2	VAL39	l	ARG2	ARG36	l	ARG2	VAL39	l	ARG2	ARG36	l
ARG2	VAL40	l	ARG2	VAL37	l	ARG2	VAL40	l	ARG2	VAL37	l
ARG2	GLU41	l	ARG2	ASP38	l	ARG2	GLU41	l	ARG2	ASP38	l
ARG2	LEU42	m	ARG2	LEU39	l	ARG2	LEU42	l	ARG2	LEU39	l
						ARG2	LYS52	m			
			ARG2	VAL52	m	ARG2	GLU55	m	ARG2	VAL52	l
ARG2	PRO67	s									
ARG2	HIS68	m				ARG2	HIS68	l			
			ARG2	PRO66	m				ARG2	PRO66	m
TYR3	HIS4	m				TYR3	HIS4	m	TYR3	PHE4	m
TYR3	THR5	m	TYR3	ILE5	m	TYR3	THR5	m	TYR3	ILE5	l
TYR3	THR6	l	TYR3	THR6	l	TYR3	THR6	l	TYR3	THR6	l
TYR3	ALA7	l				TYR3	ALA7	l			
TYR3	THR8	l				TYR3	THR8	l			
TYR3	SER9	l	TYR3	PRO7	l	TYR3	SER9	l	TYR3	PRO7	l

TYR3	PRO10	l	TYR3	ASP8	l	TYR3	PRO10	l	TYR3	ASP8	l
TYR3	ILE11	l	TYR3	ILE9	l	TYR3	ILE11	l	TYR3	ILE9	l
TYR3	SER12	m	TYR3	THR10	m	TYR3	SER12	m	TYR3	THR10	l
			TYR3	LYS12	m				TYR3	LYS12	l
TYR3	ARG25	m									
						TYR3	MET27	m	TYR3	THR24	m
TYR3	TRP28	l	TYR3	TRP25	l	TYR3	TRP28	l	TYR3	TRP25	l
TYR3	ARG36	l	TYR3	ARG33	l				TYR3	ARG33	s
TYR3	VAL39	l	TYR3	ARG36	l	TYR3	VAL39	l	TYR3	ARG36	l
TYR3	VAL40	l	TYR3	VAL37	l	TYR3	VAL40	l	TYR3	VAL37	l
TYR3	GLU41	l	TYR3	ASP38	l	TYR3	GLU41	l	TYR3	ASP38	l
TYR3	LEU42	l	TYR3	LEU39	l	TYR3	LEU42	l	TYR3	LEU39	l
TYR3	PRO67	l									
TYR3	HIS68	l	TYR3	PHE65	l	TYR3	HIS68	l	TYR3	PHE65	l
TYR3	PRO69	l	TYR3	PRO66	l	TYR3	PRO69	l	TYR3	PRO66	l
TYR3	LYS70	l	TYR3	THR67	m	TYR3	LYS70	l	TYR3	THR67	l
TYR3	GLN71	l				TYR3	GLN71	l	TYR3	ARG68	m
TYR3	ARG72	m	TYR3	LYS69	m						
TYR3	PRO73	l									
TYR3	GLY74	m									
TYR4	THR6	m	TYR4	THR6	m	TYR4	THR6	l			
TYR4	ILE11	m	TYR4	ILE9	l	TYR4	ILE11	m	TYR4	ILE9	l
TYR4	MET27	l	TYR4	THR24	l	TYR4	MET27	m	TYR4	THR24	m
TYR4	TRP28	l	TYR4	TRP25	l	TYR4	TRP28	m	TYR4	TRP25	m
TYR4	CYS29	l	TYR4	CYS26	l	TYR4	CYS29	m	TYR4	CYS26	l
TYR4	ASP30	l	TYR4	ASP27	l	TYR4	ASP30	m	TYR4	ASP27	l
TYR4	ALA31	l	TYR4	ALA28	l	TYR4	ALA31	m	TYR4	ALA28	l
TYR4	PHE32	l	TYR4	PHE29	l				TYR4	PHE29	l
TYR4	CYS33	l	TYR4	CYS30	l	TYR4	CYS33	m	TYR4	CYS30	l
TYR4	SER34	l	TYR4	SER31	l				TYR4	SER31	m
TYR4	SER35	m	TYR4	ILE32	l	TYR4	SER35	m	TYR4	ILE32	l
TYR4	ARG36	l	TYR4	ARG33	l	TYR4	ARG36	l	TYR4	ARG33	l
TYR4	GLY37	l	TYR4	GLY34	l	TYR4	GLY37	l	TYR4	GLY34	l
TYR4	LYS38	l	TYR4	LYS35	l	TYR4	LYS38	l	TYR4	LYS35	l
TYR4	VAL39	l	TYR4	ARG36	l	TYR4	VAL39	l	TYR4	ARG36	l
TYR4	VAL40	l	TYR4	VAL37	l	TYR4	VAL40	l	TYR4	VAL37	l
TYR4	GLU41	m	TYR4	ASP38	m						
			TYR4	LEU39	m				TYR4	LEU39	m
TYR4	PRO67	m									
TYR4	HIS68	l				TYR4	HIS68	l			
TYR4	PRO69	l	TYR4	PRO66	l	TYR4	PRO69	l	TYR4	PRO66	l
TYR4	LYS70	m	TYR4	THR67	m	TYR4	LYS70	m	TYR4	THR67	m
TYR4	GLN71	m	TYR4	ARG68	m	TYR4	GLN71	m	TYR4	ARG68	m
			TYR4	LYS69	m				TYR4	LYS69	m
TYR4	PRO73	m	TYR4	ARG70	m				TYR4	ARG70	l
			TYR4	PRO71	l				TYR4	PRO71	s
						GLU5	THR6	m			
			GLU5	ILE9	m				GLU5	ILE9	l
			GLU5	THR22	m				GLU5	THR22	l
GLU5	ARG25	l				GLU5	ARG25	m			
GLU5	MET27	l	GLU5	THR24	l	GLU5	MET27	l	GLU5	THR24	l
GLU5	TRP28	l	GLU5	TRP25	l	GLU5	TRP28	m	GLU5	TRP25	l
GLU5	CYS29	m	GLU5	CYS26	s	GLU5	CYS29	m			
						GLU5	ASP30	m			
			GLU5	PHE29	m				GLU5	PHE29	m
GLU5	CYS33	l	GLU5	CYS30	l	GLU5	CYS33	m	GLU5	CYS30	l
GLU5	SER34	m	GLU5	SER31	m	GLU5	SER34	m	GLU5	SER31	m
						GLU5	SER35	m			
GLU5	ARG36	l	GLU5	ARG33	l	GLU5	ARG36	l	GLU5	ARG33	l
GLU5	GLY37	l	GLU5	GLY34	l	GLU5	GLY37	l	GLU5	GLY34	l
GLU5	LYS38	l	GLU5	LYS35	l	GLU5	LYS38	l	GLU5	LYS35	l
GLU5	VAL39	l	GLU5	ARG36	l	GLU5	VAL39	l	GLU5	ARG36	l
GLU5	VAL40	l	GLU5	VAL37	l	GLU5	VAL40	l	GLU5	VAL37	l
GLU5	GLU41	m							GLU5	ASP38	m

			GLU5	LEU39	l	GLU5	LEU42	m	GLU5	LEU39	l
GLU5	GLU56	m									
GLU5	PRO67	m	GLU5	PRO64	l				GLU5	PRO64	l
GLU5	HIS68	l	GLU5	PHE65	l	GLU5	HIS68	l	GLU5	PHE65	l
GLU5	PRO69	l	GLU5	PRO66	l	GLU5	PRO69	l	GLU5	PRO66	l
GLU5	LYS70	l	GLU5	THR67	l	GLU5	LYS70	l	GLU5	THR67	l
									GLU5	ARG68	m
GLU5	GLN71	l	GLU5	ARG68	l	GLU5	GLN71	l	GLU5	LYS69	l
			GLU5	ARG70	m				GLU5	ARG70	m
			GLU5	PRO71	s						
						SER6	MET27	m			
			SER6	PHE29	m				SER6	PHE29	s
SER6	ARG25	s									
SER6	MET27	m									
SER6	CYS33	m							SER6	CYS30	m
SER6	SER34	m	SER6	SER31	m				SER6	SER31	m
SER6	SER35	l	SER6	ILE32	l	SER6	SER35	m	SER6	ILE32	l
SER6	ARG36	l	SER6	ARG33	l	SER6	ARG36	l	SER6	ARG33	l
SER6	GLY37	l	SER6	GLY34	l	SER6	GLY37	m	SER6	GLY34	l
SER6	LYS38	l	SER6	LYS35	l	SER6	LYS38	l	SER6	LYS35	l
SER6	VAL39	l	SER6	ARG36	m	SER6	VAL39	l	SER6	ARG36	m
SER6	VAL40	l	SER6	VAL37	l	SER6	VAL40	l	SER6	VAL37	l
			SER6	PRO64	m				SER6	PRO64	m
SER6	HIS68	l	SER6	PHE65	m	SER6	HIS68	l	SER6	PHE65	m
SER6	PRO69	l	SER6	PRO66	l	SER6	PRO69	l	SER6	PRO66	l
SER6	LYS70	l	SER6	THR67	l	SER6	LYS70	l	SER6	THR67	l
SER6	GLN71	l	SER6	ARG68	l	SER6	GLN71	l	SER6	ARG68	l
			SER6	LYS69	l	SER6	ARG72	m	SER6	LYS69	l
			SER6	ARG70	l				SER6	ARG70	l
			SER6	PRO71	l				SER6	PRO71	l
SER7	PHE32	l	SER7	PHE29	l				SER7	PHE29	m
SER7	SER35	l	SER7	ILE32	l	SER7	SER35	m	SER7	ILE32	l
SER7	ARG36	l	SER7	ARG33	l	SER7	ARG36	l	SER7	ARG33	l
SER7	GLY37	l	SER7	GLY34	l	SER7	GLY37	m	SER7	GLY34	l
SER7	LYS38	l	SER7	LYS35	m	SER7	LYS38	m	SER7	LYS35	l
SER7	VAL39	m	SER7	ARG36	m	SER7	VAL39	m			
SER7	VAL40	m	SER7	VAL37	l	SER7	VAL40	m	SER7	VAL37	m
SER7	HIS68	l				SER7	HIS68	l			
SER7	PRO69	l	SER7	PRO66	l	SER7	PRO69	l	SER7	PRO66	l
SER7	LYS70	l	SER7	THR67	l	SER7	LYS70	l	SER7	THR67	l
SER7	GLN71	l	SER7	ARG68	l	SER7	GLN71	m	SER7	ARG68	l
			SER7	LYS69	l				SER7	LYS69	l
			SER7	ARG70	l				SER7	ARG70	l
			SER7	PRO71	l				SER7	PRO71	l
LEU8	HIS4	m	LEU8	PHE4	m	LEU8	HIS4	m			
LEU8	THR6	m	LEU8	THR6	m	LEU8	THR6	l	LEU8	THR6	m
			LEU8	PRO7	m	LEU8	SER9	m	LEU8	PRO7	m
			LEU8	ASP8	l	LEU8	PRO10	m	LEU8	ASP8	l
LEU8	ILE11	m	LEU8	ILE9	l	LEU8	ILE11	l	LEU8	ILE9	l
			LEU8	THR10	m				LEU8	THR10	m
LEU8	ARG36	l	LEU8	ARG33	l	LEU8	ARG36	m	LEU8	ARG33	l
									LEU8	GLY34	m
LEU8	VAL40	m	LEU8	VAL37	l	LEU8	VAL40	l	LEU8	VAL37	l
LEU8	LEU42	m	LEU8	LEU39	l	LEU8	LEU42	m	LEU8	LEU39	l
LEU8	ASN66	m									
LEU8	PRO67	m									
LEU8	HIS68	l	LEU8	PHE65	l	LEU8	HIS68	l	LEU8	PHE65	l
LEU8	PRO69	l	LEU8	PRO66	l	LEU8	PRO69	l	LEU8	PRO66	l
LEU8	LYS70	l	LEU8	THR67	l	LEU8	LYS70	l	LEU8	THR67	l
LEU8	GLN71	l	LEU8	ARG68	l	LEU8	GLN71	l	LEU8	ARG68	l
LEU8	ARG72	l	LEU8	LYS69	l	LEU8	ARG72	l	LEU8	LYS69	l
LEU8	PRO73	m	LEU8	ARG70	l				LEU8	ARG70	l

LEU8	GLY74	m	LEU8	PRO71	l				LEU8	PRO71	m
						GLU9	SER9	m			
			LEU9	ASP8	m						
LEU9	ILE11	m	LEU9	ILE9	l	GLU9	ILE11	m	GLU9	ILE9	m
LEU9	PHE32	m	LEU9	PHE29	m				GLU9	PHE29	m
LEU9	ARG36	l	LEU9	ARG33	l	GLU9	ARG36	l	GLU9	ARG33	l
									GLU9	GLY34	m
			LEU9	ARG36	m	GLU9	VAL39	l	GLU9	ARG36	l
						GLU9	VAL40	m	GLU9	VAL37	m
LEU9	HIS68	l				GLU9	HIS68	l			
			LEU9	PRO66	l				GLU9	PRO66	l
LEU9	LYS70	m							GLU9	THR67	m
LEU9	GLN71	l	LEU9	ARG68	m				GLU9	ARG68	l
			LEU9	LYS69	l				GLU9	LYS69	m
			LEU9	ARG70	l	GLU9	LYS70	l	GLU9	ARG70	m
			LEU9	PRO71	l	GLU9	GLN71	m	GLU9	PRO71	m
PRO10	THR6	l	PRO10	THR6	l	PRO10	THR6	l	PRO10	THR6	l
PRO10	ALA7	s				PRO10	ALA7	m			
PRO10	THR8	m				PRO10	THR8	l			
PRO10	SER9	l	PRO10	PRO7	l	PRO10	SER9	l	PRO10	PRO7	l
PRO10	PRO10	l	PRO10	ASP8	l	PRO10	PRO10	m	PRO10	ASP8	l
PRO10	ILE11	l	PRO10	ILE9	l	PRO10	ILE11	l	PRO10	ILE9	l
PRO10	ARG36	m				PRO10	ARG36	m	PRO10	ARG33	m
PRO10	VAL39	m	PRO10	ARG36	m	PRO10	VAL39	l	PRO10	ARG36	m
			PRO10	PRO66	m				PRO10	PRO66	m
PRO10	HIS68	l				PRO10	HIS68	m			
PRO10	GLN71	l				PRO10	GLN71	m	PRO10	ARG68	m
PRO10	ARG72	m	PRO10	LYS69	l				PRO10	LYS69	m
PRO10	PRO73	m							PRO10	ARG70	s
PRO10	GLY74	m	PRO10	PRO71	m						
TYR11	THR6	m	TYR11	THR6	m	TYR11	THR6	l	TYR11	THR6	m
						TYR11	THR8	l			
TYR11	SER9	m	TYR11	PRO7	m				TYR11	PRO7	m
						TYR11	TRP28	l	TYR11	TRP25	l
						TYR11	ASP30	m	TYR11	ASP27	l
						TYR11	ALA31	m	TYR11	ALA28	l
TYR11	PHE32	l	TYR11	PHE29	l				TYR11	PHE29	l
TYR11	ARG36	l	TYR11	ARG33	l	TYR11	ARG36	l	TYR11	ARG33	l
						TYR11	GLY37	l			
						TYR11	LYS38	m			
TYR11	VAL39	l	TYR11	ARG36	l	TYR11	VAL39	l	TYR11	ARG36	l
			TYR11	VAL37	m	TYR11	VAL40	l	TYR11	VAL37	l
						TYR11	GLU41	l	TYR11	ASP38	m
TYR11	HIS68	m				TYR11	HIS68	m			
			TYR11	PRO66	m						
TYR11	GLN71	m									
			TYR11	LYS69	m						
			TYR11	ARG70	m						
			TYR11	PRO71	l						
						PRO12	THR8	l			
PRO12	SER9	l	PRO12	PRO7	m				PRO12	PRO7	l
PRO12	ARG36	l	PRO12	ARG33	m						
			PRO12	ARG36	m				PRO12	ARG36	l

						PRO12	VAL39	l			
ASP13	ALA7	m									
ASP13	THR8	s				ASP13	THR8	m			
ASP13	SER9	m									
ASP13	TRP28	m	ASP13	TRP25	m	ASP13	TRP28	l	ASP13	TRP25	l
ASP13	ARG36	m									
ASP13	VAL39	m	ASP13	ARG36	l	ASP13	VAL39	m	ASP13	ARG36	l
			ASP13	ASP38	m	ASP13	GLU41	l	ASP13	ASP38	l

* All the contacts in the table “aligned” considering αBgt-αCtx and HAP-HAP[L9E] amino acid sequence alignment (Fig 2A, 3A). Each contact in the αBgt-HAP complex is put in correspondence with analogous contacts in the complexes αCtx-HAP, αBgt-HAP[L9E], and αCtx-HAP[L9E]. In case when an analogous contact was not observed during MD, the corresponding cell in the table is empty.

** Lifetime of each contact counted as part of MD-trajectory total time (200ns). “s” stands for short-living contacts: lifetime is less than 10 %; “m” stands for medium-living contacts: lifetime is less than 50 %; “l” – long stands for long-living contacts: lifetime is greater than 50 %.

Section 2

PGCA application for three-component solution spectra

Presented results aim to demonstrate PGCA applicability to determination of complexation constant using the spectra of three-component solutions and the influence of various factors on fitting results. The solution components are analyte A, titrant T and their complex C, which interacts according to chemical reaction equation A + T <=> C. We demonstrate that PGCA method is fully applicable to systems both with well resolved spectra and systems with similar spectra of different components. The 1% error in the experimental intensities yields ~10% error of complexation constant in case of well resolved spectra and ~50% error in case of overlapping peaks for 10 samples titration series. Complexation constant error decreases with experimental intensities error reduction and the increase of sample number in titration series. Three-component PGCA yields acceptable values of complexation constant even in case of non-luminescent complex (with zero quantum yield).

Brief method description

Background for PGCA method and its application to fluorescence and absorption spectra of 2-(2′-hydroxyphenyl) benzimidazole is described in details in Al-Soufi et al (2001)[1] Here we will give a brief summary of the method. Experimental spectra are presented as a matrix **Y** with size n_s x n_w, where n_s is a number of spectra and n_w is a number of wavelengths. It is used to obtain covariation matrix $\mathbf{Y}^T\mathbf{Y}$. Eigenvalues λ of covariation matrix can be used to estimate the number of components in the system. Another index for components number estimation is a mean residual error σ weighted by the remaining degrees of freedom:

$$\sigma_k^2 = \frac{\sum_{l=k+1}^{n_s} \lambda_l}{(n_s - k)(n_w - k)}$$

The eigenvectors **V** of covariation matrix are abstract spectra and corresponding eigenvalues **Z** are their contribution to experimental spectra. The number of components n_c is 3 for all our simulations. Eigenvalues $\tilde{\mathbf{Z}}$ corresponding to n_c highest eigenvalues are used for least-squares fitting using the

1Al-Soufi, W., Novo, M., & Mosquera, M. (2001). Principal component global analysis of fluorescence and absorption spectra of 2-(2′-hydroxyphenyl) benzimidazole. Applied Spectroscopy, 55(5), 630-636.

chemical reaction model. Concentrations of each component at each solution **D** are functions of equilibrium constants and known total reagent concentration **D** = H(K; x). In our case **D** is a solution of a following equation system:

$$K=\frac{[C]}{[A][T]}$$
$$[A]_{tot}=[A]+[C]$$
$$[T]_{tot}=[T]+[C]$$

The following residual χ^2 is minimized to obtain optimal equilibrium constants:

$$\chi^2=\sum_{i=1}^{n_s}\sum_{l=1}^{n_c}\left(\left(\tilde{\mathbf{Z}}-\mathbf{D}\left(\mathbf{D}^{+}\tilde{\mathbf{Z}}\right)\right)_{il}\right)^2$$

Eigenvalues are connected with components concentrations by the following relation:

$$\tilde{\mathbf{Z}}=\mathbf{D}\mathbf{P} \quad (1)$$

“Rotation” matrix **P** is estimated as $\mathbf{P}=\mathbf{D}^{+}\tilde{\mathbf{Z}}$. $\mathbf{D}^{+}$ is a pseudoinverse of **D**

Further we will provide numerical simulations to evaluate this approach for complexation constants determination.

Basic case

As a basic case we will consider the system of analyte, titrant and complex with well resolved spectra (Fig. 1A). The concentrations are $[A]_{tot}$ = 1, $[B]_{tot}$ = 0—1, titration series contains N = 11 spectra including the titrant spectra (Fig. 1B), true value of complexation constant is K = 1, and the initial fitting value K_{init} = 100.

Plots of covariation matrix indexes suggests that three components are presented as there are three points over the noise level for each plot (Fig. 1C, 1D). Fit matches the modeled concentration dependency (Fig 1E) and the obtained value of complexation constant K_{est} = 0.999999999999 +/- $1.38 \cdot 10^{-13}$ corresponds to true value K = 1.

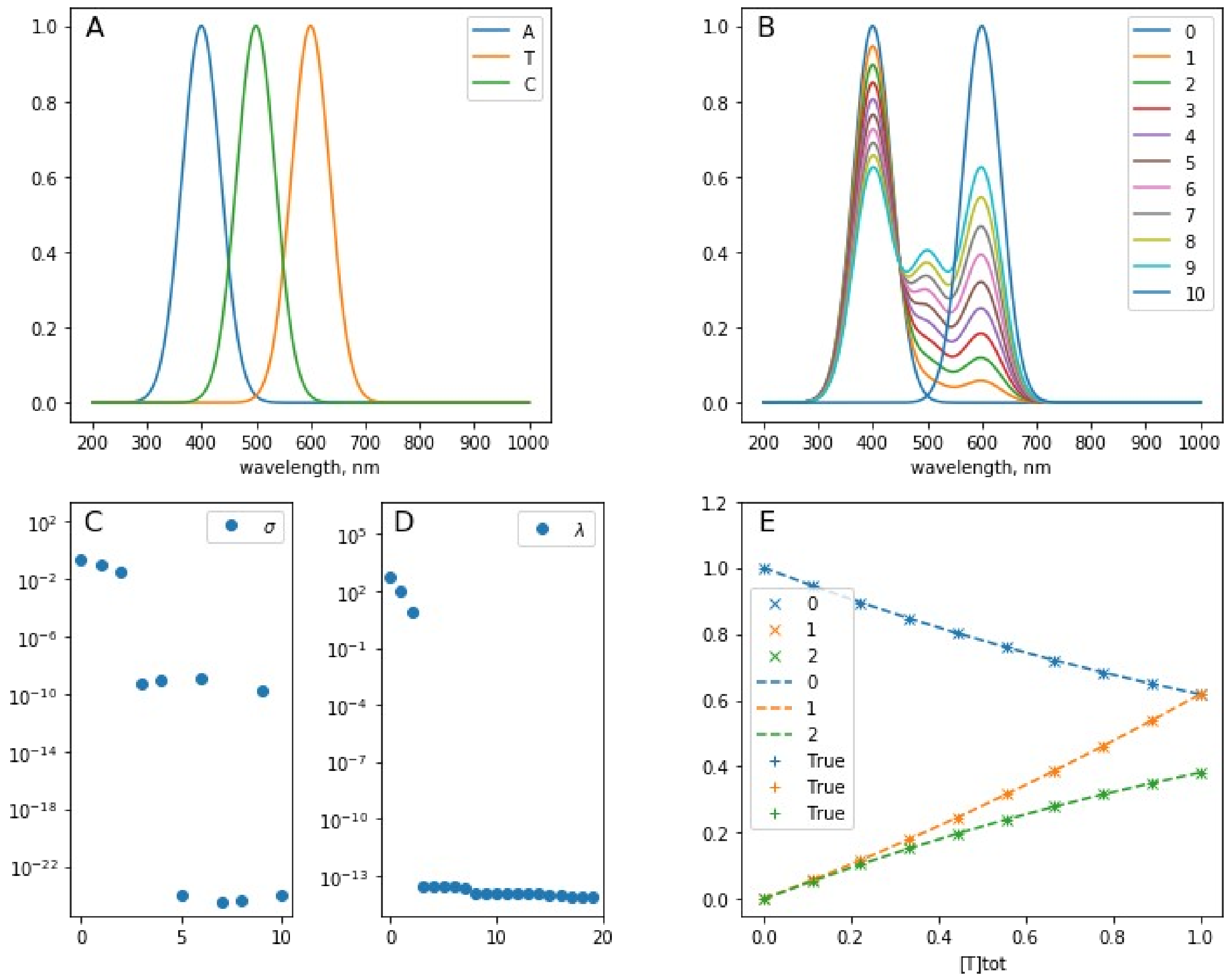

Figure S1. A Components spectra of the simulation . B. Simulated titration spectra. C, D. Indexes of covariation matrix. E. True titration concentrations (plus symbol), components concentrations fit obtained using optimal K value (dashed line) and components concentration estimation obtained using eigenvalues and the rotation matrix for optimal K (see eq. 1) (x-cross symbol).

Overlapping spectra case.

Consider analyte, titrant and complex system with highly overlapping spectra with the similar parameter values: $[A]_{tot} = 1$, $[B]_{tot} = 0—1$, $N = 11$, $K = 1$, $Ki_{nit} = 100$. This case yield similarly good fit (Fig. 2E) and $K_{est} = 0.9999999999991127 +/- 1.59\cdot10^{-13}$.

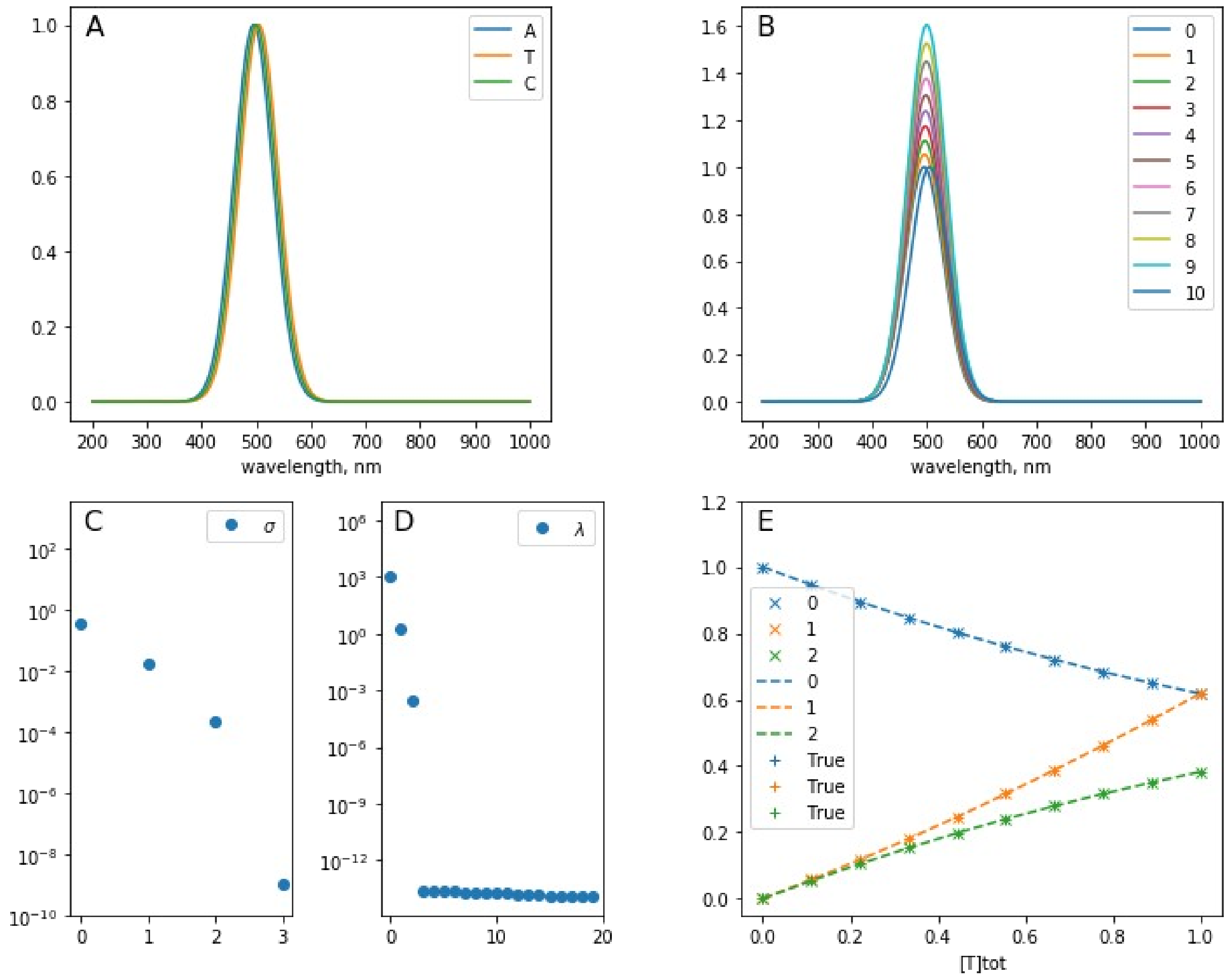

Figure S2. A Components spectra of the simulation . B. Simulated titration spectra. C, D. Indexes of covariation matrix. E. True titration concentrations (plus symbol), components concentrations fit obtained using optimal K value (dashed line) and components concentration estimation obtained using eigenvalues and the rotation matrix for optimal K (see eq. 1) (x-cross symbol).

Basic case with noise

Consider the case with well resolved spectra of components in presence of additive experimental noise with standard deviation equal to 1% of spectra maxima with the model parameters corresponding to basic case. This scenario leads to the estimated value of complexation constant

Kest = 1.19 +/- 0.10.

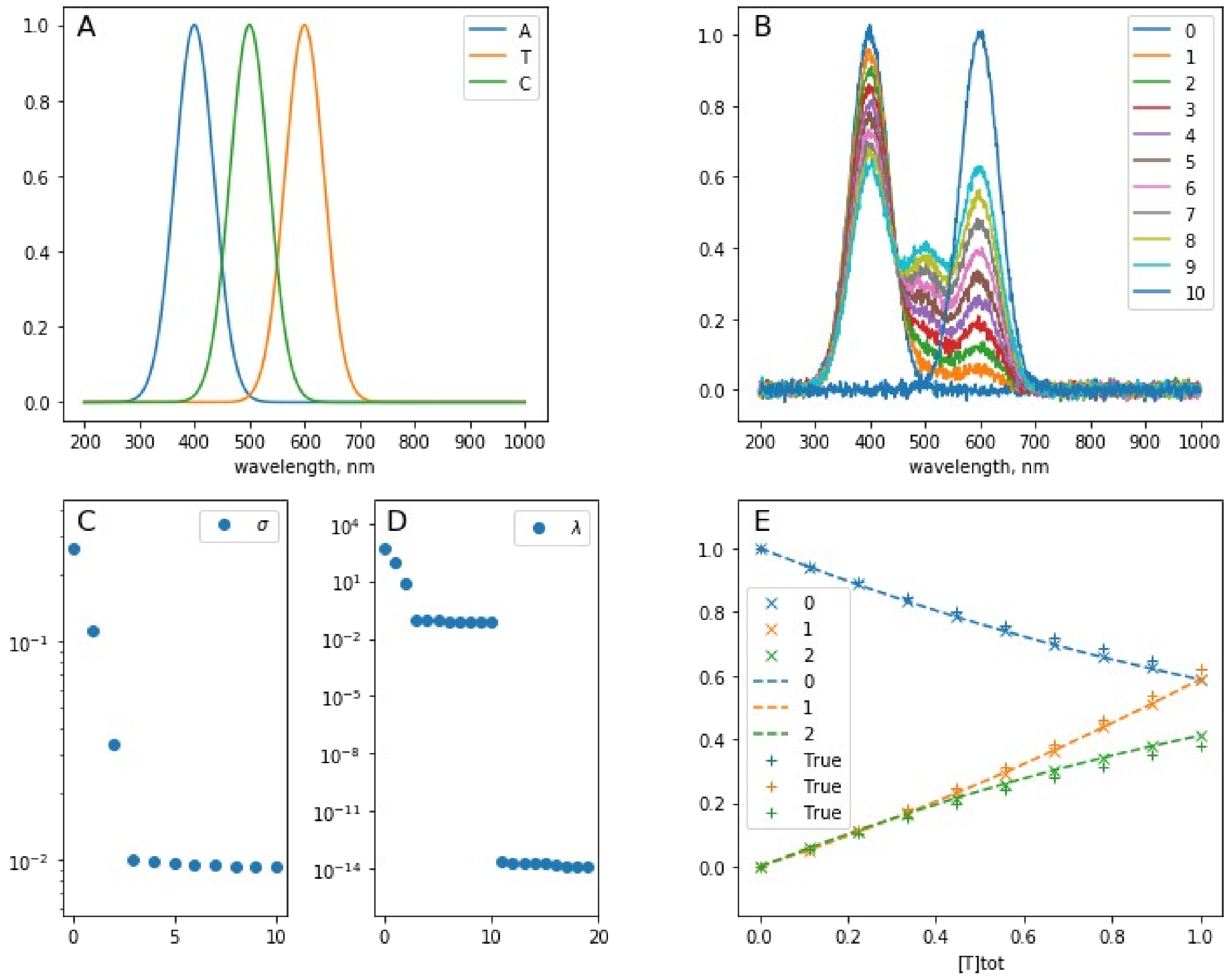

Figure S3. A Components spectra of the simulation . B. Simulated titration spectra. C, D. Indexes of covariation matrix. E. True titration concentrations (plus symbol), components concentrations fit obtained using optimal K value (dashed line) and components concentration estimation obtained using eigenvalues and the rotation matrix for optimal K (see eq. 1) (x-cross symbol).

Basic case with noise and increased titration samples number

The complexation constant error can be reduced with the increase of sample number in case of same noise intensity. Consider sample number N = 31 with the same parameter values as in the previous case. This case leads to the estimated value Kest = 0.96 +/- 0.05 with the error reduction factor close to square root of sample numbers ratio. Thus estimation error can be reduced by extending the titration series. The corresponding plots are presented at Fig. 4.

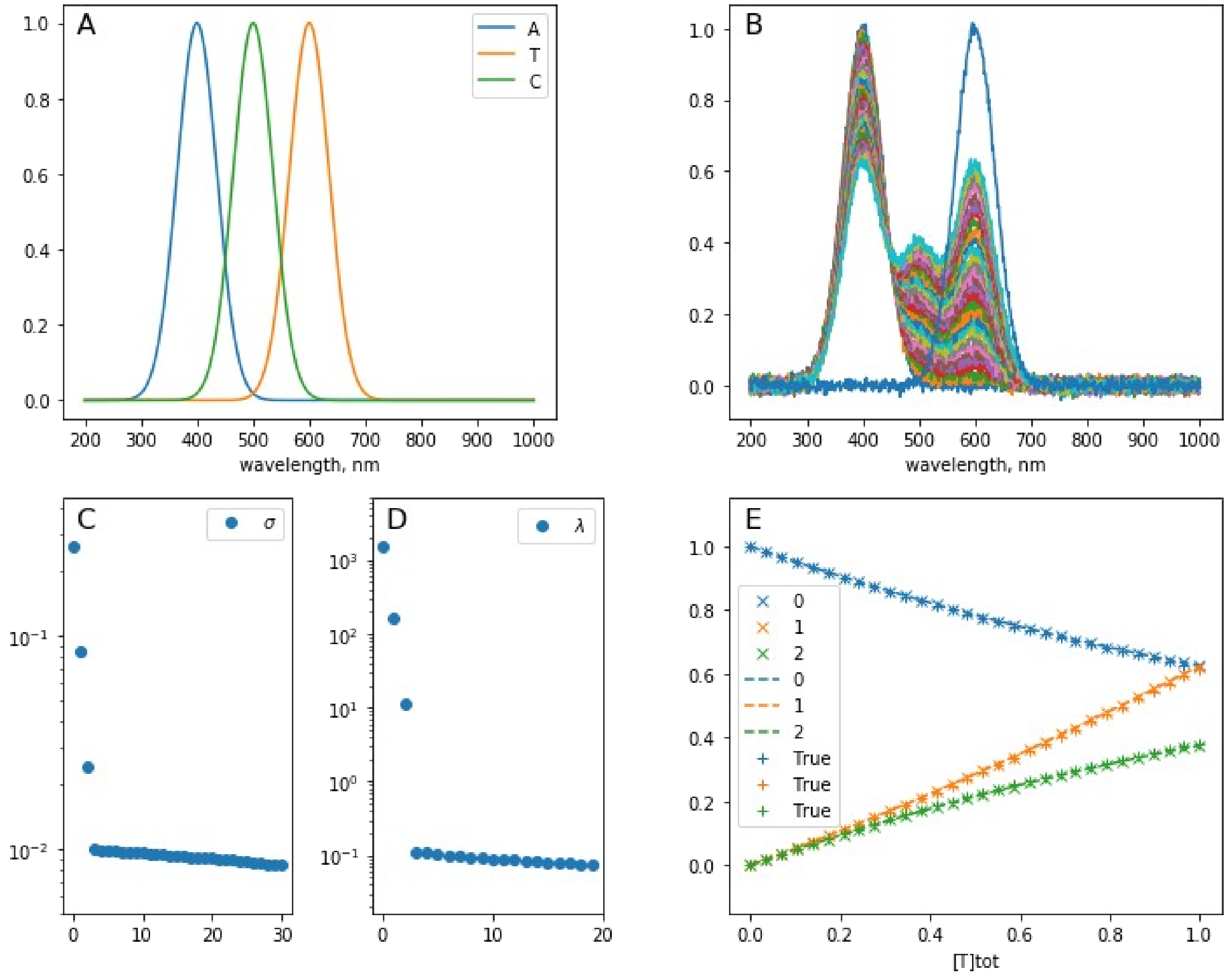

Figure S4 A Components spectra of the simulation . B. Simulated titration spectra. C, D. Indexes of covariation matrix. E. True titration concentrations (plus symbol), components concentrations fit obtained using optimal K value (dashed line) and components concentration estimation obtained using eigenvalues and the rotation matrix for optimal K (see eq. 1) (x-cross symbol).

Overlapping components in presence of noise

Consider the case with the similar spectra of anlyte, titrant and complex in the presence of experimental noise (~1%) (Fig. 5A, 5B). Spectra of titration series are very similar in presence of noise. Correlation indexes plots (Fig 5C, 5D) demonstrate two index values greater than the noise level. This leads to the fact that third component eigenvalues become very noisy (Fig 6). Never the less three-component fit of obtained projections leads to the complexation constant estimation K_{est} = 0.85 +/- 0.59 and acceptable fitting curve profile (dashed curves at Fig 5E). The mean value of K_{est} is close to the real value K = 1. Thus it can be used for the order of magnitude estimation despite the high value of estimated variance. Transformation of noisy projections to concentrations using the relation from eq. 1. leads to noisy estimation of concentrations (x-crosses at Fig 5E) and should not be taken into account in this case.

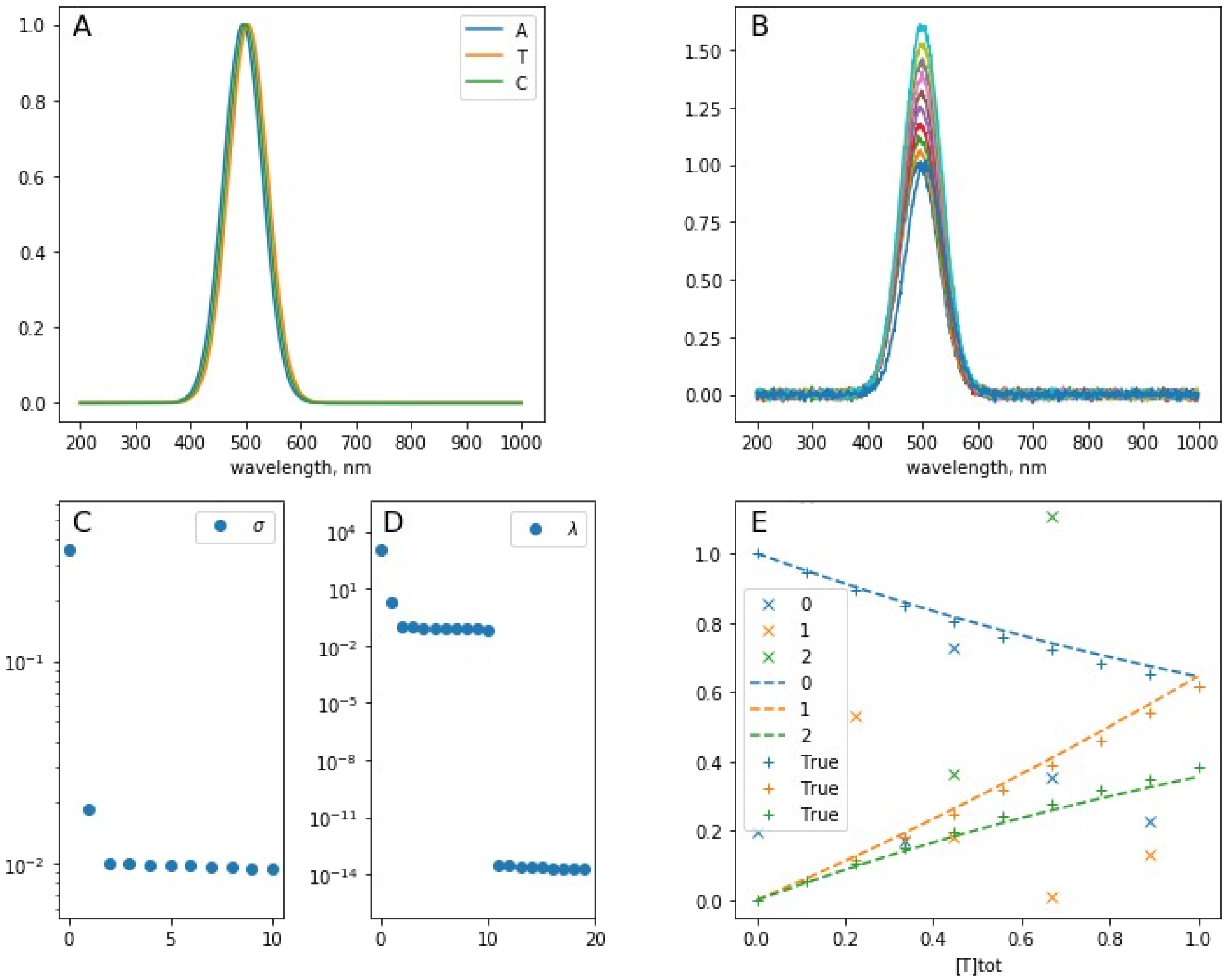

Figure S5 A Components spectra of the simulation . B. Simulated titration spectra. C, D. Indexes of covariation matrix. E. True titration concentrations (plus symbol), components concentrations fit

obtained using optimal K value (dashed line) and components concentration estimation obtained using eigenvalues and the rotation matrix for optimal K (see eq. 1) (x-cross symbol).

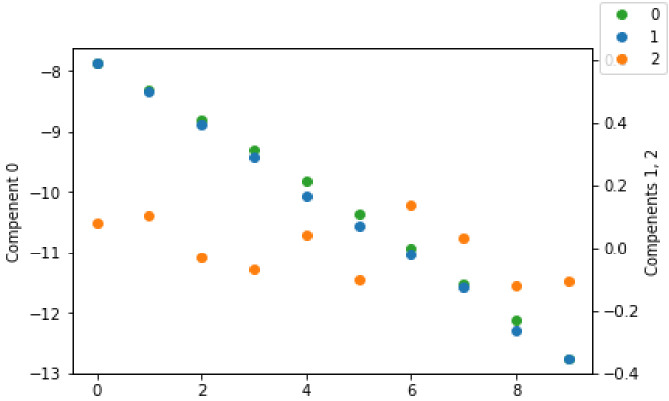

Figure S6. Eigenvalues corresponding to first three components (indexing from 0). It can be seen that the third component (denoted “2” at the figure) becomes noisy in the current case.

Overlapping components in presence of noise continued

Samples number increase leads to decreased value of estimation error. For sample number **N = 30** K_{est} = 0.95 +/- 0.29. Thus, 3 fold sample number increase leads to two fold error decrease. It should be noted that components number estimation using Fig 7C, 7D leads to the assumption of 2 detected components. This value differs from true value n_c = 3. The concentrations obtained by eigenvalues transformation still deviate from true concentrations values due to reasons discussed earlier, but the fitting curve profile matches to simulated concentrations used for fitting.

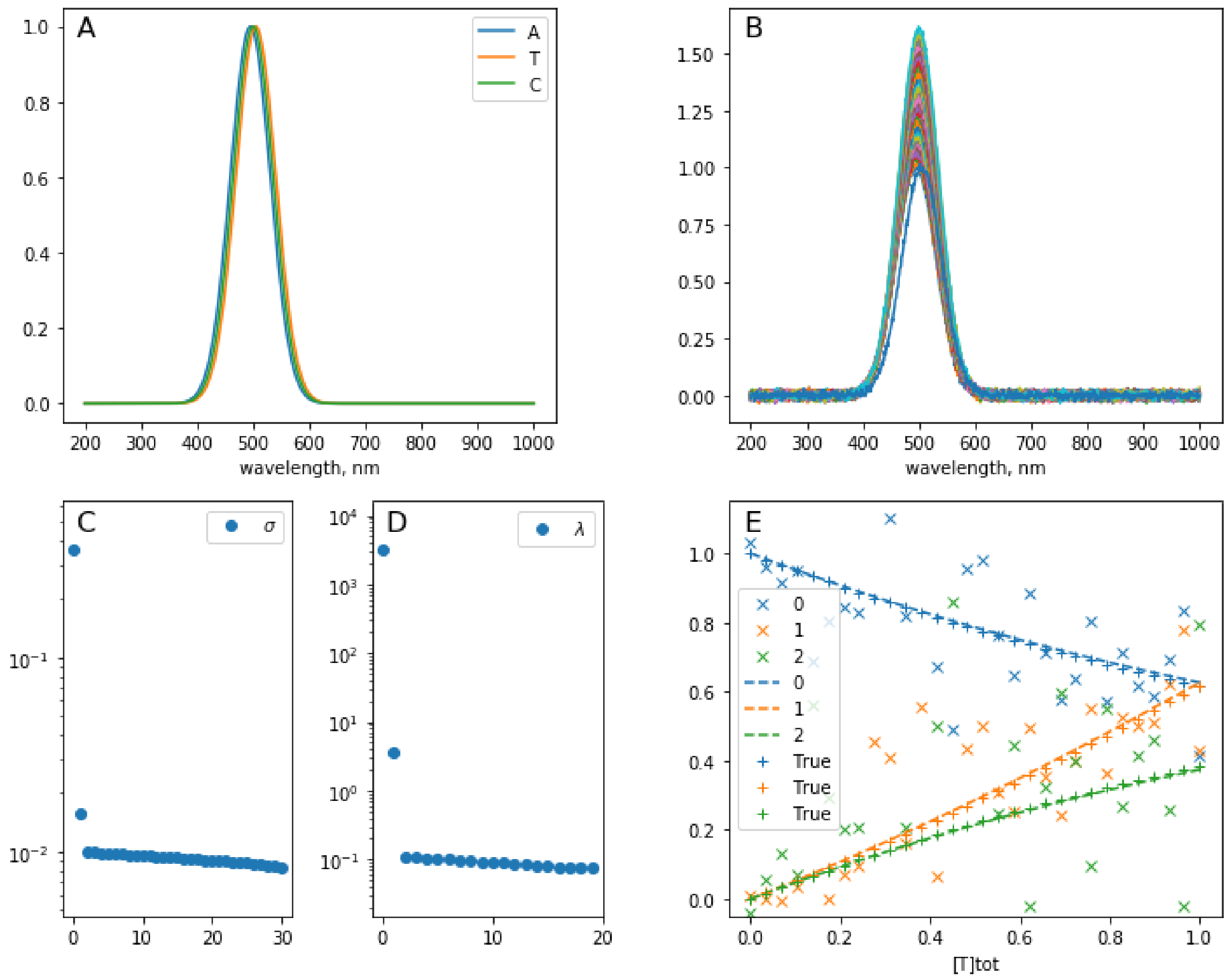

Figure S7. A Components spectra of the simulation . B. Simulated titration spectra. C, D. Indexes of covariation matrix. E. True titration concentrations (plus symbol), components concentrations fit obtained using optimal K value (dashed line) and components concentration estimation obtained using eigenvalues and the rotation matrix for optimal K (see eq. 1) (x-cross symbol).

Overlapping components in presence of noise continued

Similar complexation constant error decrease can be obtained by the experimental noise reduction.

Three fold noise reduction (0.003% of spectra amplitude) yields two-fold error reduction: K_{est} = 1.02 +/- 0.24. Figs. 8C, 8D, 8E demonstrate similar problems described in previous paragraph.

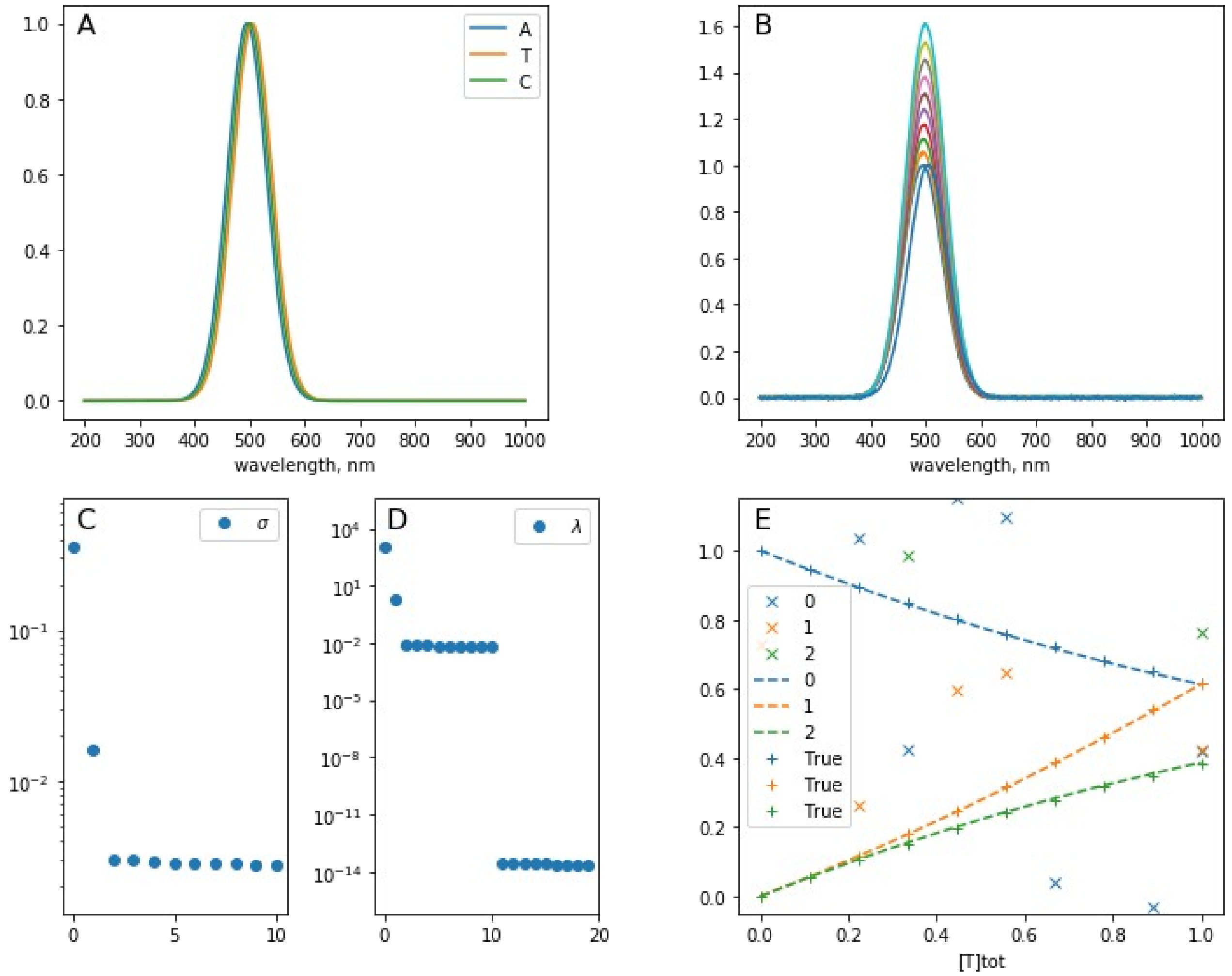

Figure S8. A Components spectra of the simulation . B. Simulated titration spectra. C, D. Indexes of covariation matrix. E. True titration concentrations (plus symbol), components concentrations fit obtained using optimal K value (dashed line) and components concentration estimation obtained using eigenvalues and the rotation matrix for optimal K (see eq. 1) (x-cross symbol).

Nonfluorescent complex

It should be noted that in case of non-fluorescent complex (Fig 9A) and the same parameters as in previous case, complexation constant still can be obtained using the same three-component fitting method: K_{est} = 1.00 +/- 0.11.

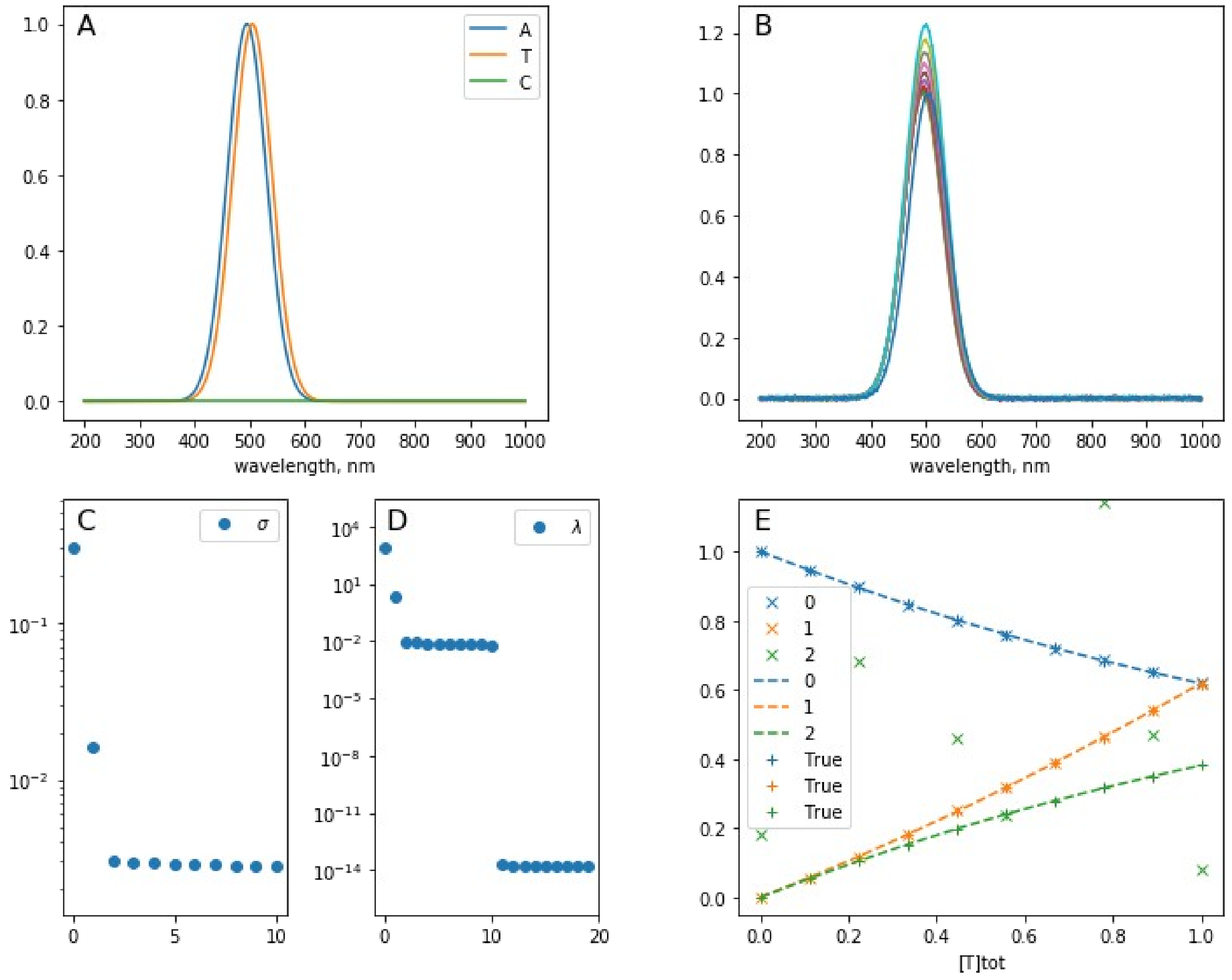

Figure S9. A Components spectra of the simulation . B. Simulated titration spectra. C, D. Indexes of covariation matrix. E. True titration concentrations (plus symbol), components concentrations fit obtained using optimal K value (dashed line) and components concentration estimation obtained using eigenvalues and the rotation matrix for optimal K (see eq. 1) (x-cross symbol).

Section 3

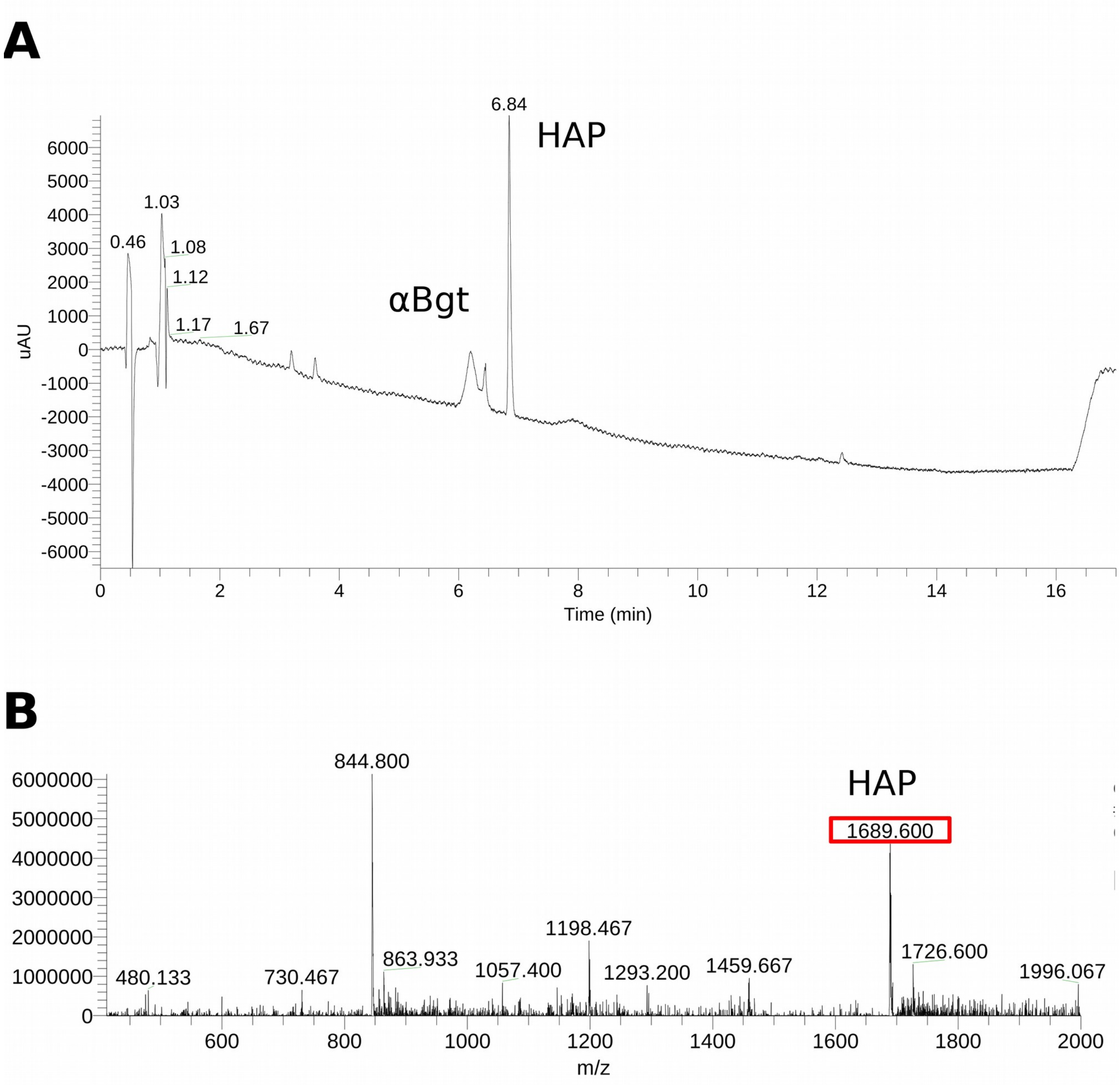

Figure S10. LC-MS of the first peak from gel-filtration of HAP-αBgt mixture. **A**: Reverse-phase chromatogram of the first peak reveals two major components. The wide double peak is attributed to αBgt (consisting of two isoforms). The narrow peak is attributed to HAP; **B**: Mass-spectrum of the narrow peak from the reverse-phase chromatogram. HAP m/z peak is highlighted.

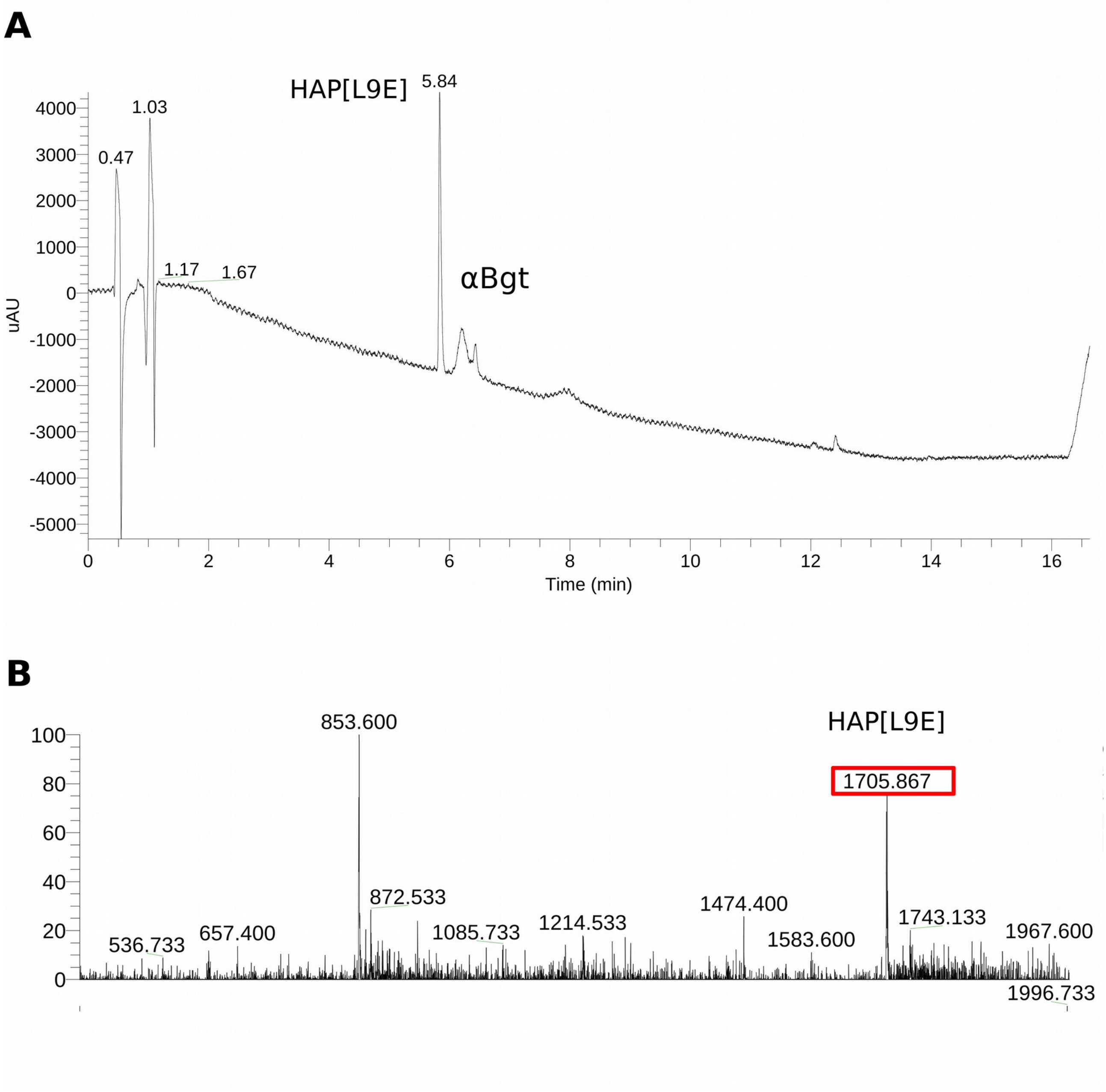

Figure S11. LC-MS of the first peak from gel-filtration of HAP[L9E]-αBgt mixture. **A**: Reverse-phase chromatogram of the first peak reveals two major components. The wide double peak is attributed to αBgt (consisting of two isoforms). The narrow peak is attributed to HAP[L9E]. Note that HAP[L9E] elutes earlier than HAP (Fig. S9) showing more hydrophilic nature due to L9E substitution; **B**: Mass-spectrum of the narrow peak from the reverse-phase chromatogram. HAP[L9E] m/z peak is highlighted.

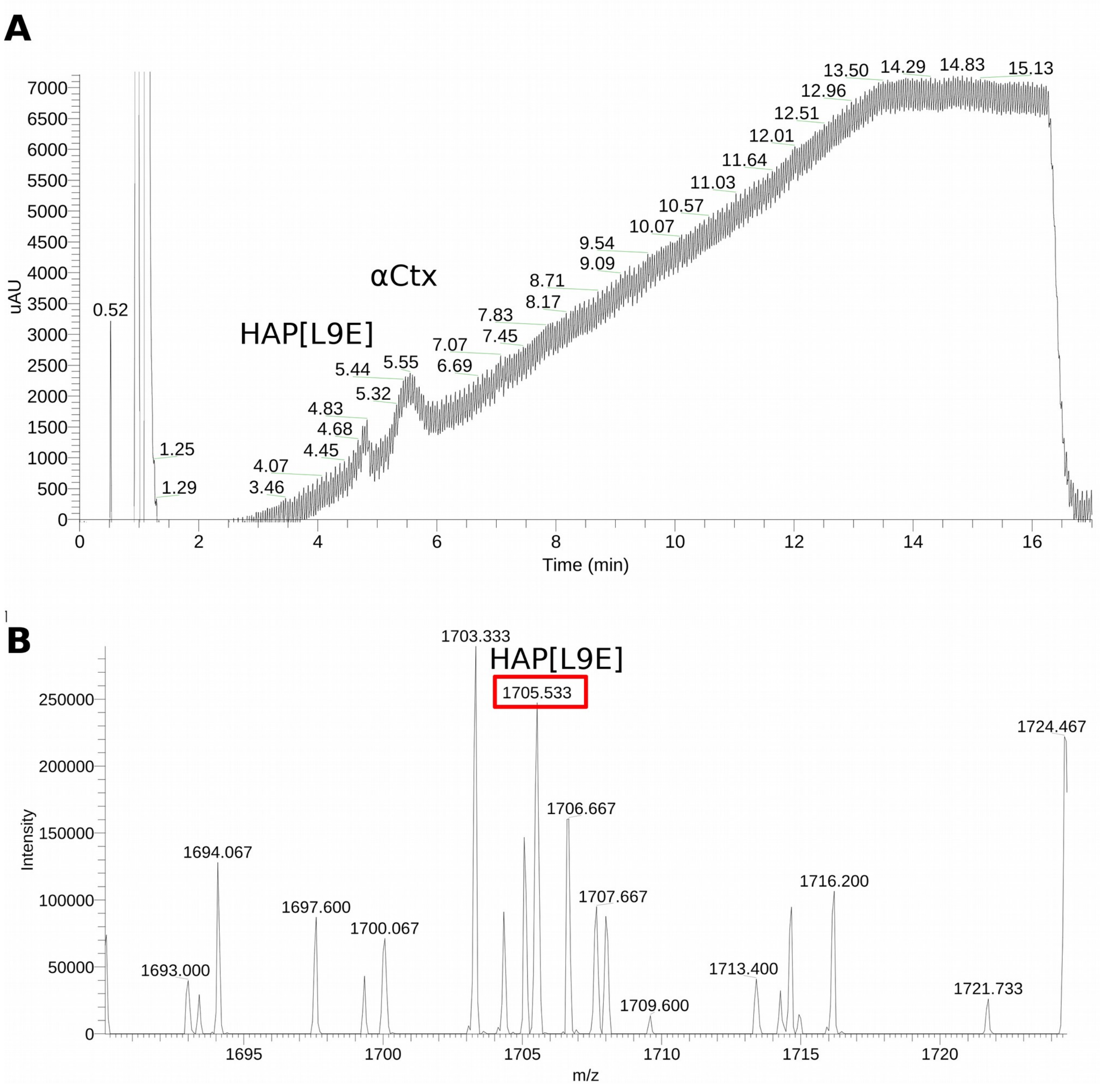

Figure S12. LC-MS of the first peak from gel-filtration of HAP[L9E]-αCtx mixture. **A**: Reverse-phase chromatogram of the first peak reveals two major components. The first peak is attributed to HAP[L9E], the second peak is αCtx; **B**: Mass-spectrum of the first peak from the reverse-phase chromatogram. HAP[L9E] m/z peak is highlighted.

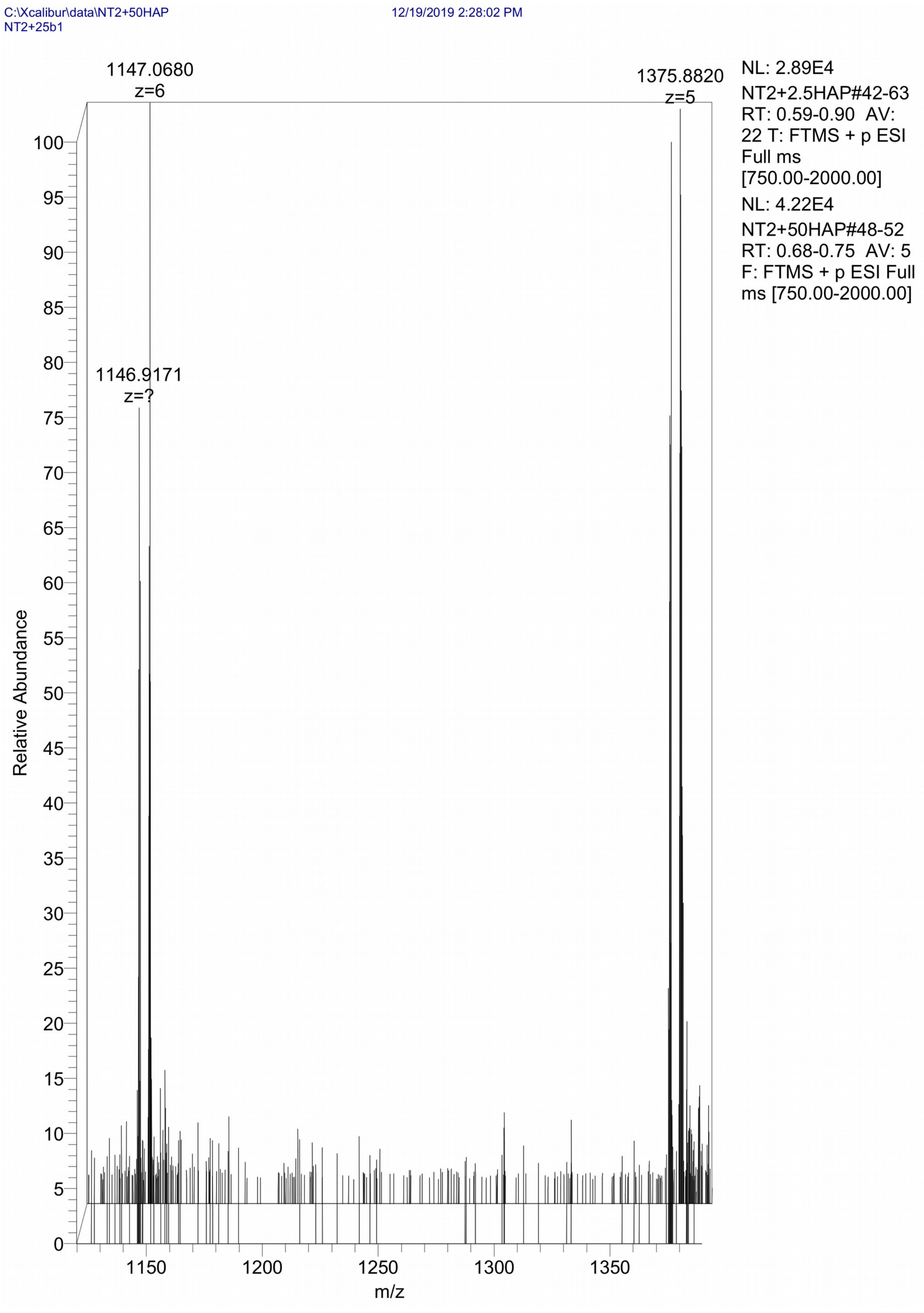

Figure S13. HAP does not bind to NT2 short α-neurotoxin (MW 6880). Native ESI MS shows no signs of m/z corresponding to HAP-NT2 complex.

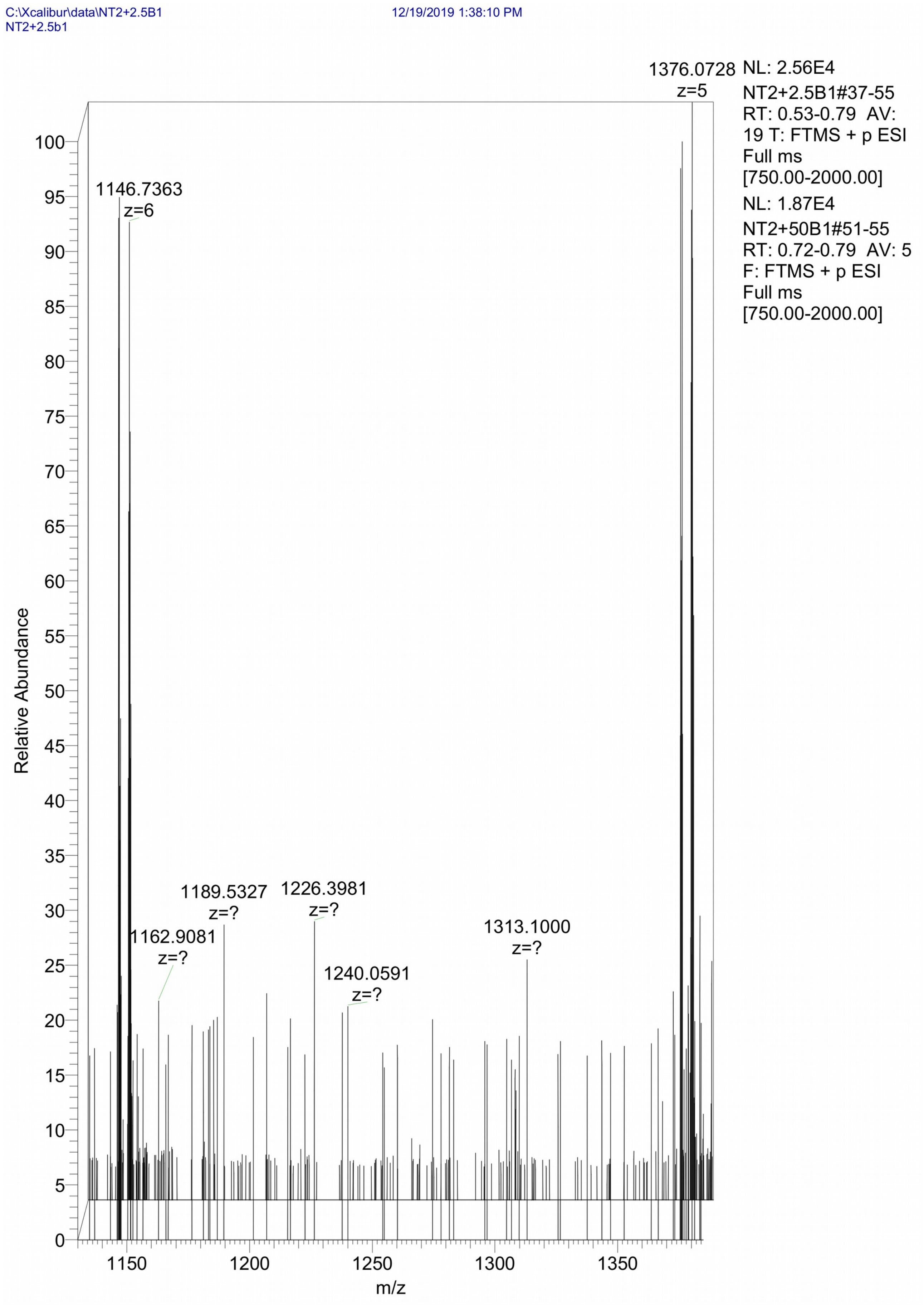

Figure S14. HAP[L9E] does not bind to NT2 short α-neurotoxin (MW 6880). Native ESI MS shows no signs of m/z corresponding to HAP[L9E]-NT2 complex.
